# Supplementary material for: Chromatin accessibility of circulating CD8+ T cells predicts treatment response to PD-1 blockade in patients with gastric cancer
Source: Nat Commun. 2021 Feb 12;12:975. doi: 10.1038/s41467-021-21299-w (PMC7881150; doi:10.1038/s41467-021-21299-w)
Supplement: Supplementary file 1 — Supplementary Information [file 41467_2021_21299_MOESM1_ESM.pdf]

# Supplementary Information

## Chromatin accessibility of circulating CD8+ T cells predicts treatment response to PD-1 blockade in patients with gastric cancer

Hyun Mu Shin[1,2,6*†], Gwanghun Kim[2,3,6†], Sangjib Kim[8†], Ji Hyun Sim[3†], Jiyeob Choi[2,4], Minji Kim[2,3,6], Minsuk Kwon[10], Sang-Kyu Ye[2,5,6,7], Dong-Sup Lee[2,3,6,7], Seung Woo Cho[9], Seung Tae Kim[10], Jeeyun Lee[10*], and Hang-Rae Kim[1,2,3,6,7*]

Affiliations:
[1]Wide River Institute of Immunology, Seoul National University, Hongcheon 25159, Republic of Korea
[2]Department of Biomedical Sciences, Seoul National University College of Medicine, Seoul 03080, Republic of Korea
[3]Department of Anatomy and Cell Biology, Seoul National University College of Medicine, Seoul 03080, Republic of Korea
[4]Department of Preventive Medicine, Seoul National University College of Medicine, Seoul 03080, Republic of Korea
[5]Department of Pharmacology, Seoul National University College of Medicine, Seoul 03080, Republic of Korea
[6]BK21 FOUR Biomedical Science Project, Seoul National University College of Medicine, Seoul 03080, Republic of Korea
[7]Medical Research Institute, Seoul National University College of Medicine, Seoul 03080, Republic of Korea
[8]Department of Mathematics, College of Science, Korea University, Seoul 02841, Republic of Korea.
[9]Department of Biomedical Engineering, School of Life Sciences, Ulsan National Institute of Science and Technology (UNIST), Ulsan 44919, Republic of Korea
[10]Department of Medicine, Division of Hematology-Oncology, Samsung Medical Center, Sungkyunkwan University School of Medicine, Seoul 06351, Republic of Korea

†These authors contributed equally to this work

**Supplementary Fig. 1.** Gating strategies used for flow cytometry analysis and cell sorting. **a** Gating strategy to analyze PD-1$^+$CD8$^+$ T cells and Ki-67$^+$ of PD-1$^+$CD8$^+$ T cells presented on Fig. 1b–c. **b** Gating strategy to sort CD8$^+$ T cells used on the ATAC-seq presented on Fig. 3–5.

# Supplementary Fig. 1

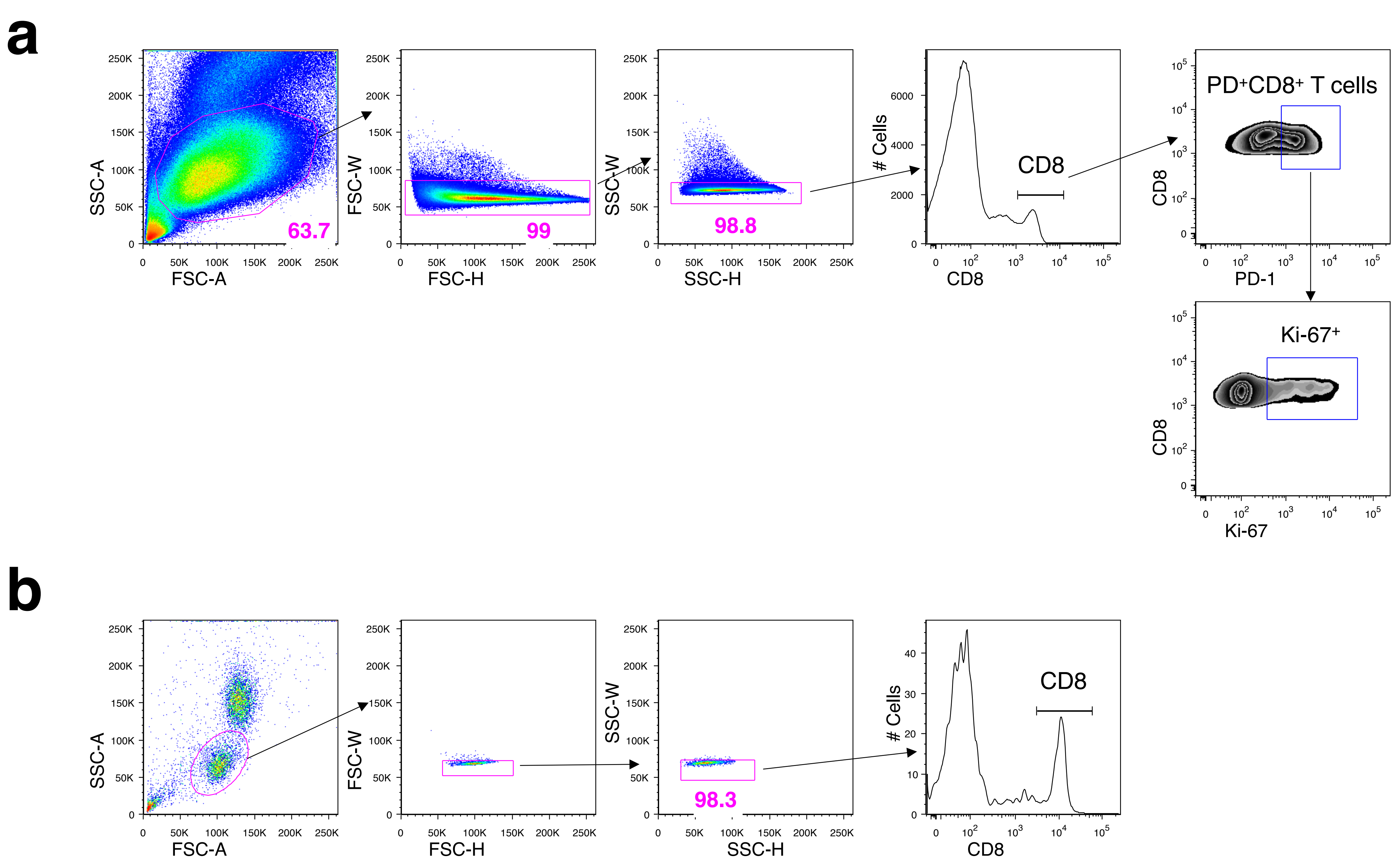

**Supplementary Fig. 2.** Genome browser snapshots of 20 selected controls. The genome browser identified 20 control peaks among previously studied PB $CD8^+$ T-cell subsets (naïve, cyan; effector memory, pink; central memory, yellow-green) and PB $CD8^+$ T cells used in this study. Numbers above peaks are control IDs. The *y*-axis shows ATAC-seq read counts. Patients with CR, PR, and PD in the responder and non-responder groups, respectively, are annotated in green and red, respectively.

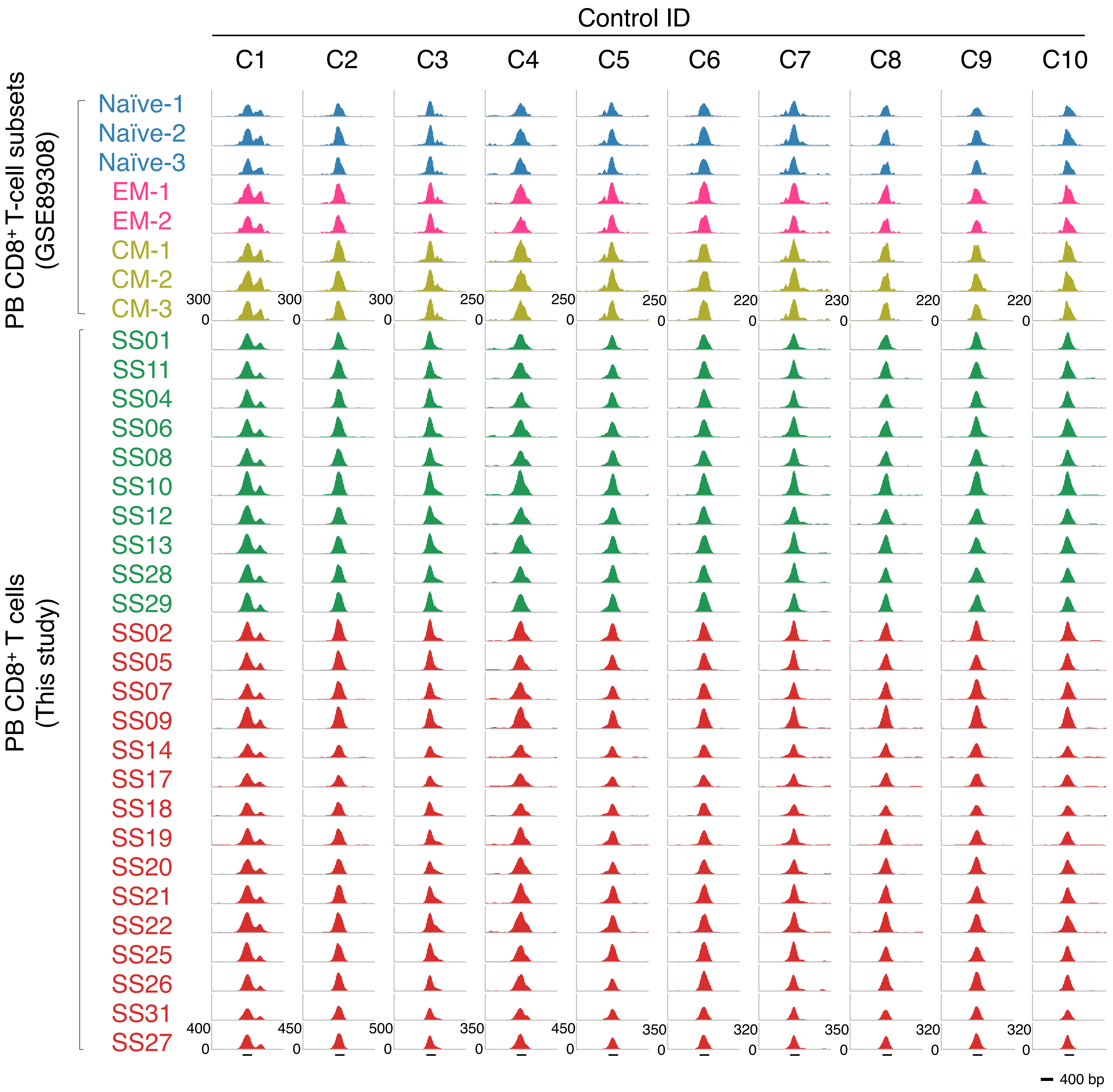
Control ID
C1
C2
C3
C4
C5
C6
C7
C8
C9
C10
PB CD8+ T-cell subsets (GSE89308)
Naïve-1
Naïve-2
Naïve-3
EM-1
EM-2
CM-1
CM-2
CM-3
300
0
300
0
300
0
250
0
250
0
250
0
220
0
230
0
220
0
220
0
PB CD8+ T cells (This study)
SS01
SS11
SS04
SS06
SS08
SS10
SS12
SS13
SS28
SS29
SS02
SS05
SS07
SS09
SS14
SS17
SS18
SS19
SS20
SS21
SS22
SS25
SS26
SS31
SS27
400
0
450
0
500
0
350
0
450
0
350
0
320
0
350
0
320
0
320
0
400 bp

Supplementary Fig. 2

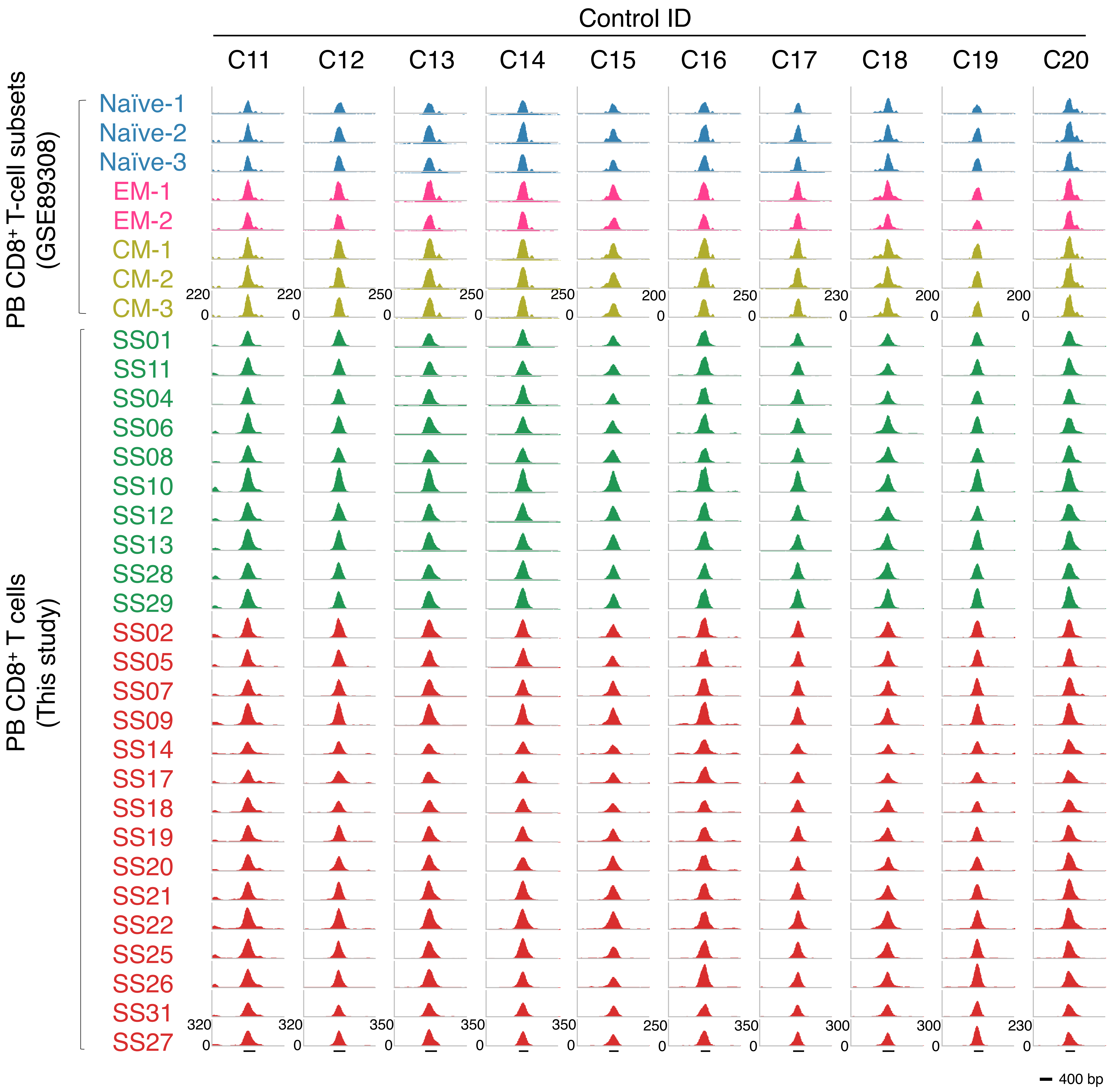

**Supplementary Fig. 3.** Universal controls in other cell types. Representatives among 20 control peaks identified using the genome browsers. Raw ATAC-seq data retrieved from the National Center for Biotechnology Information (NCBI) GEO depository site were converted into Bigwig files using the Homer suite; **a** eight hematopoietic cells, **b** three acute myeloid leukemia cells from GSE74912, normal bronchial epithelial cells, small cell lung cancer cells, normal prostate basal epithelial cells, and small cell prostate cancer cells from GSE118204, and **c** epidermal growth factor receptor (EGFR)-negative and -positive glioblastomas from GSE117685.

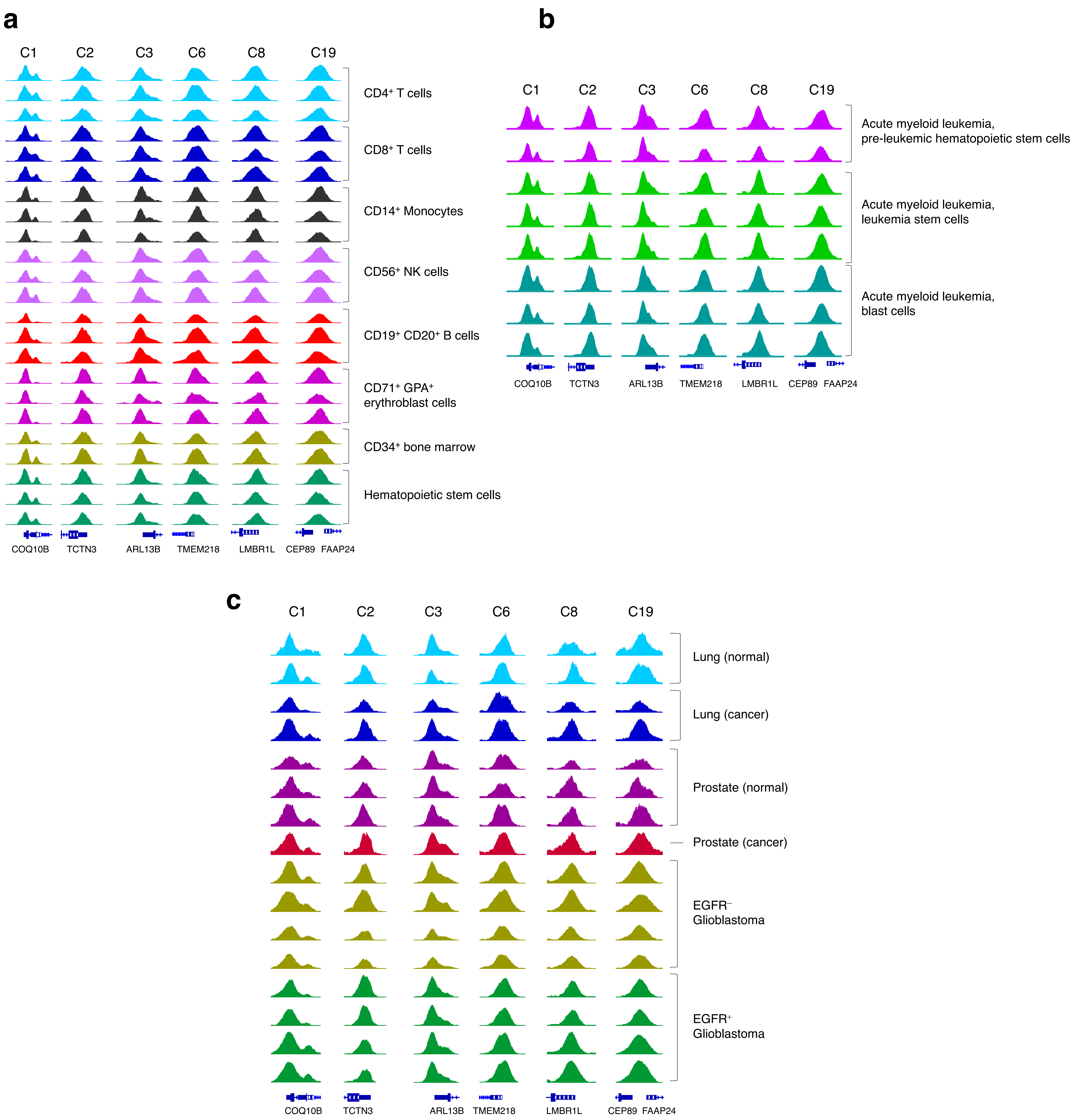
a
C1
C2
C3
C6
C8
C19
CD4+ T cells
CD8+ T cells
CD14+ Monocytes
CD56+ NK cells
CD19+ CD20+ B cells
CD71+ GPA+
erythroblast cells
CD34+ bone marrow
Hematopoietic stem cells
COQ10B
TCTN3
ARL13B
TMEM218
LMBR1L
CEP89
FAAP24
b
C1
C2
C3
C6
C8
C19
Acute myeloid leukemia,
pre-leukemic hematopoietic stem cells
Acute myeloid leukemia,
leukemia stem cells
Acute myeloid leukemia,
blast cells
COQ10B
TCTN3
ARL13B
TMEM218
LMBR1L
CEP89
FAAP24
c
C1
C2
C3
C6
C8
C19
Lung (normal)
Lung (cancer)
Prostate (normal)
Prostate (cancer)
EGFR−
Glioblastoma
EGFR+
Glioblastoma
COQ10B
TCTN3
ARL13B
TMEM218
LMBR1L
CEP89
FAAP24

**Supplementary Fig. 4.** Workflow for computing the ATAC-seq sample normalization factor. With the assumption that the shape of selected control ATAC peaks differed among samples, control ATAC peaks were converted into a simple pattern by dividing the peak area by the peak width to calculate the average height ($k$). Next, the mean height ($h$) was calculated as the average of all $k$ values for $m$ control peaks in each sample; this value represented the degree of chromatin openness among the control peaks. Finally, the normalization factor ($F$) of each sample was derived by dividing the average $h$ of all samples in a cohort by $h$ of the corresponding sample to correct the openness value of the ATAC peak.

## Computing average height

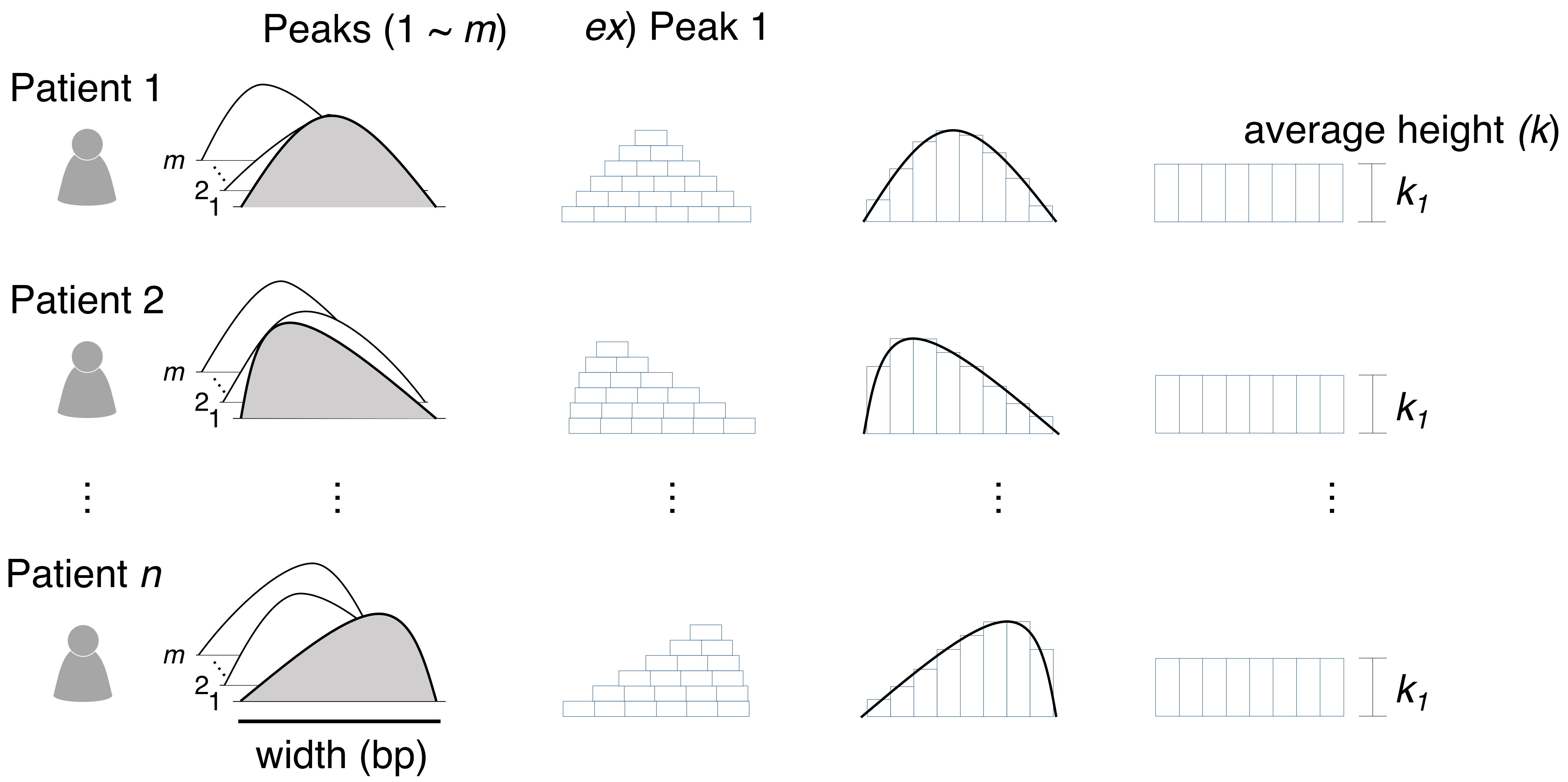


Average height ($k$) of an individual peak

$$k = \frac{area}{width} = \frac{\text{the sum of enrichment within the peak}}{\text{the base length of the peak}}$$

Mean height *(h)* of selected peaks in each sample

$$h = \frac{\sum_{i=1}^{m} k_i}{m},$$

$k_i$ = the average height of the $i$th peak in a sample

$m$ = the number of selected peaks in a sample

Normalization factor ($F$):

$$F_{ab} = \frac{S_a\left(=\frac{\sum_{i=1}^{n_a} h_{ai}}{n_a}\right)}{h_{bj}} \text{ for } 1 \le a \le b \le 2$$

$h_{ai}$ = the mean height of the $i$th sample in the cohort $C_a$

$h_{bj}$ = the mean height of the $j$th sample in the cohort $C_b$

$n_a$ = the number of samples in the cohort $C_a$

**Supplementary Fig. 5.** Selection of multiple normalization controls. The number of controls (*m*) was selected for 232 control peaks and ranked in descending order by the mean area of the sample peaks. Individual normalization factors (*F*) were then calculated as described in Supplementary Fig. 4 and Methods. **a** Pairwise variation was calculated for every series of $F_m$ and $F_{m+1}$ to reflect the effect of adding the control (*m*+1). Gray vertical lines represent the optimal numbers of controls for peak normalization among samples. **b** The 121 normalized differential peaks were ranked by mean area for all $F_m$ series; each peak is plotted to visualize changes in the rank of each peak.

Supplementary Fig. 5

**a**

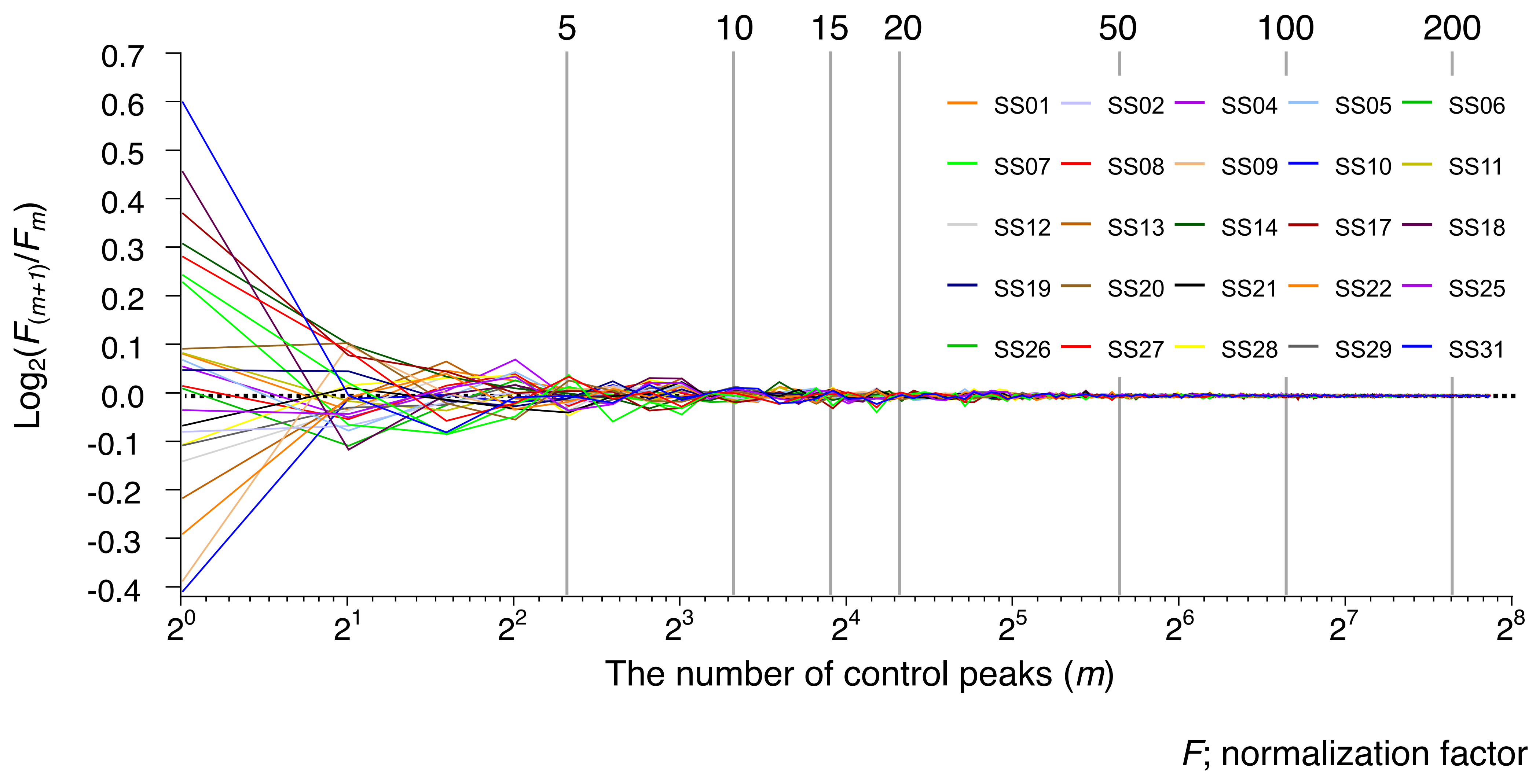


**b**

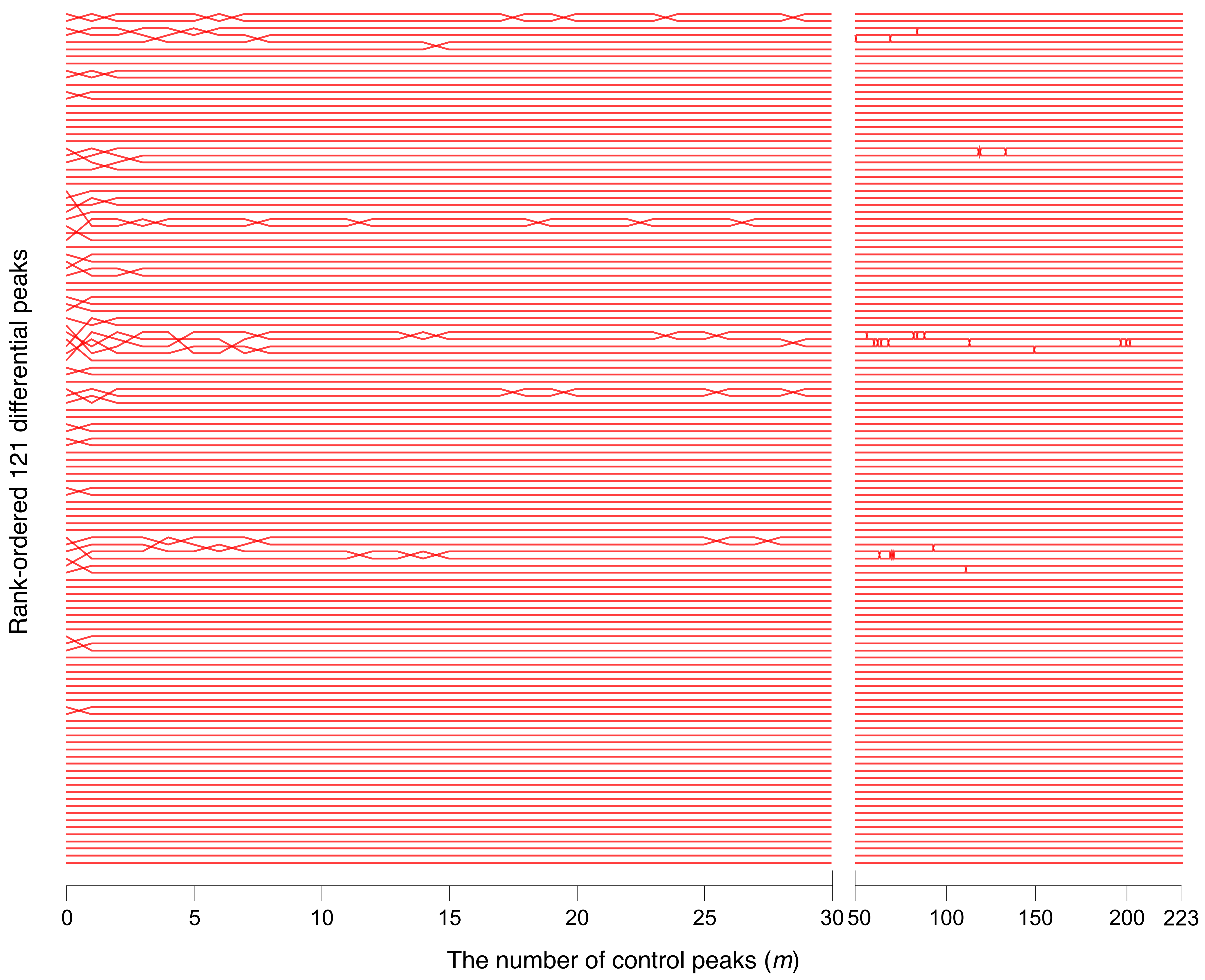

**Supplementary Fig. 6.** Comparison of differential peaks for a series of controls. Following the selection of the number of controls (*m*) as shown in Supplementary Fig. 4, normalization factors with five (red circle, $C_5$), 20 (blue circle, $C_{20}$), and 50 (black circle, $C_{50}$) controls (Supplementary Table 5) were calculated and applied to the area values of 2,560 differential peaks. After the first step in Fig. 3**a**, differential peaks for each control set were compared using a Venn diagram; 67 differential peaks were shared in results processed by two sets of controls ($C_{20}$ and $C_{50}$).

Supplementary Fig. 6

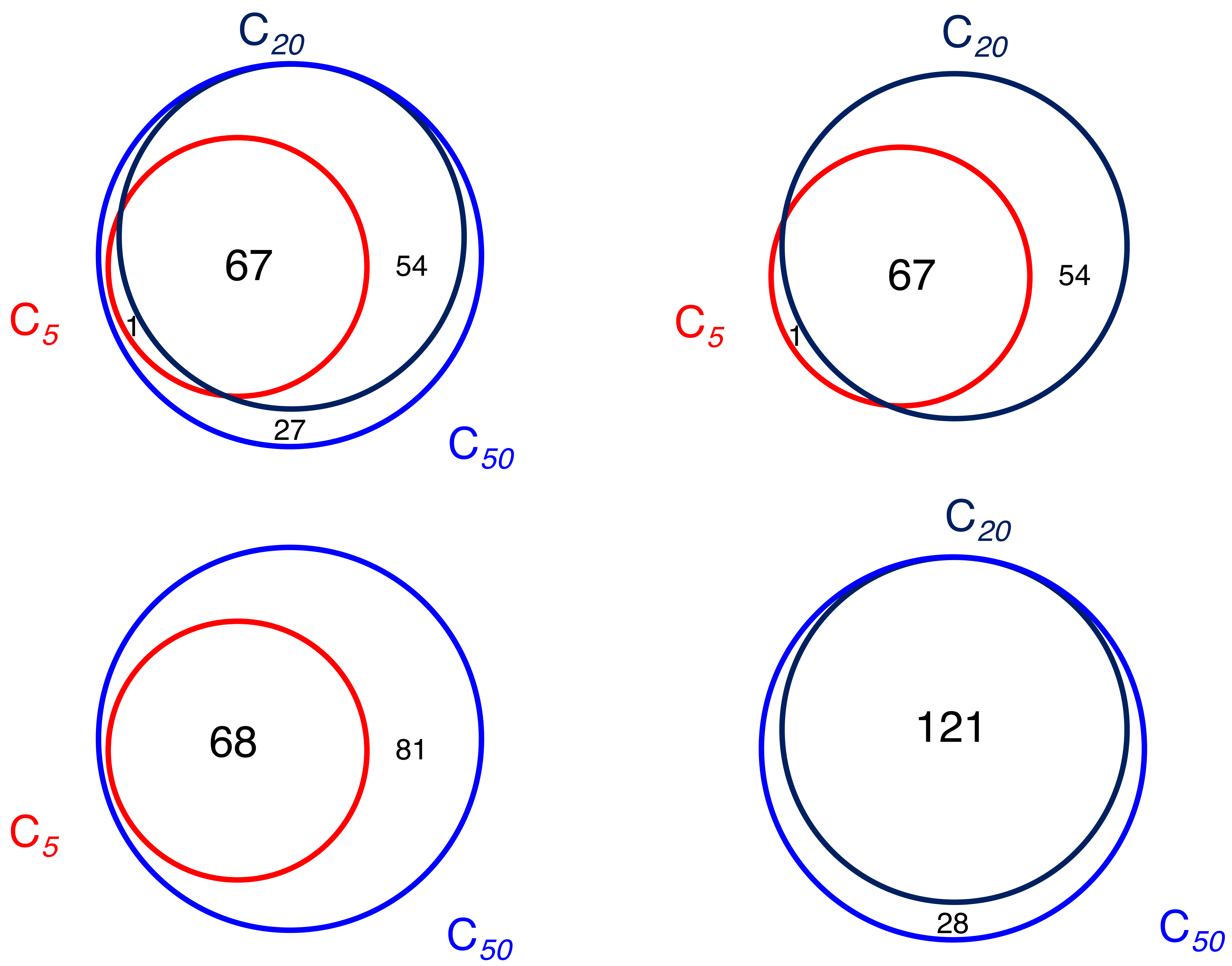

**Supplementary Fig. 7.** Peak normalization. ATAC peaks were normalized with 20 controls. **a** Pre- and post-normalized total peak enrichments (85,625, area > 1) are displayed in violin plots. Green and red boxes indicate the responder group (CR+PR) and non-responder group (PD), respectively. Hinges of all boxplots correspond to values of the 25th, 50th, and 75th percentiles, whereas boxplot whiskers extend 1st and 99th percentile. **b** In total, 2,560 differential peaks were identified using three peak callers: the HOMER suite, MACS2, and CisGenome; these were normalized by 20 control peaks. Pre- and post-normalized areas of these peaks are shown as a heatmap. Relative minimum and maximum area values are indicated in navy and yellow, respectively.

**a**

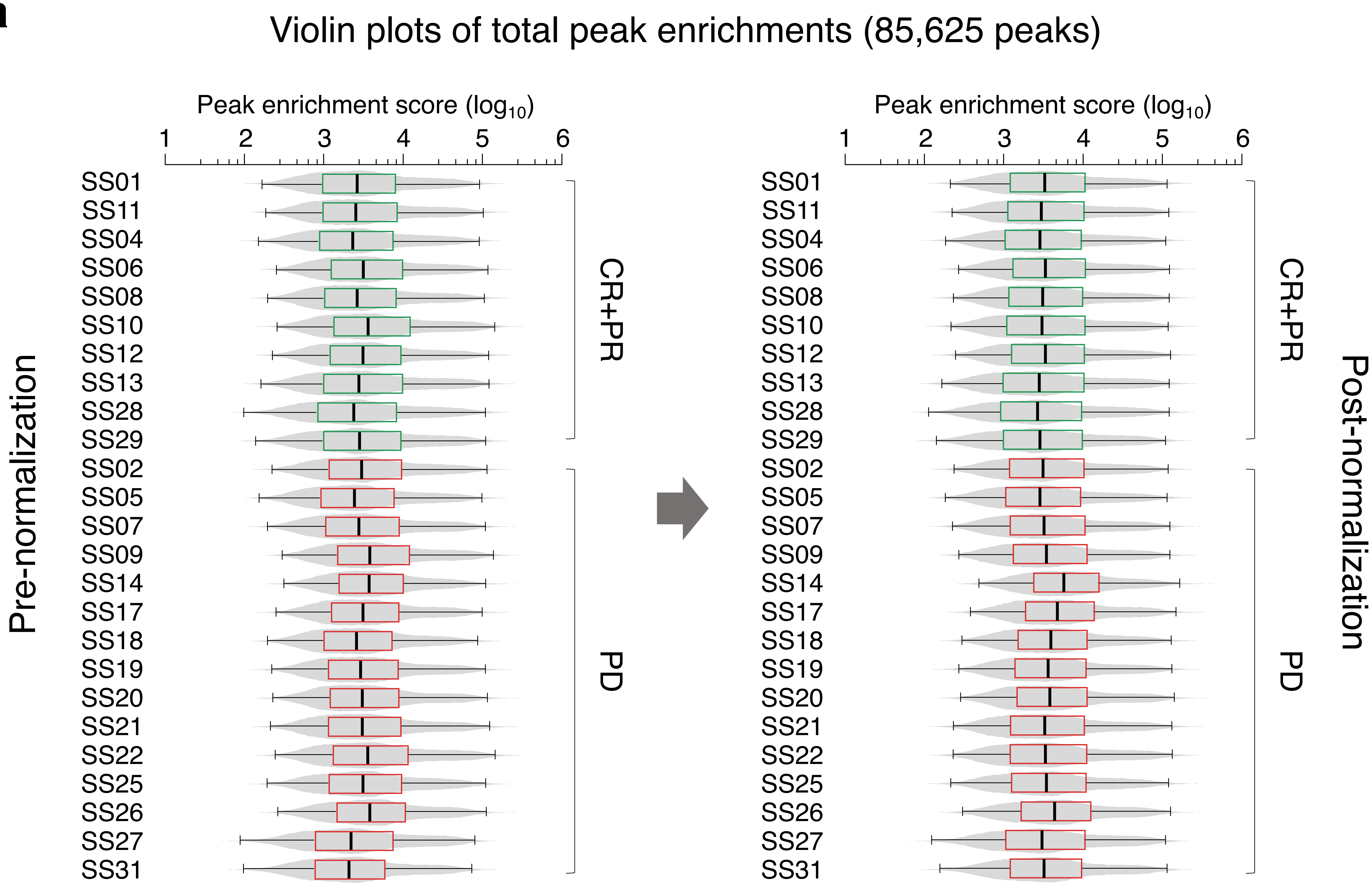
Violin plots of total peak enrichments (85,625 peaks)
Peak enrichment score (log10)
1
2
3
4
5
6
SS01
SS11
SS04
SS06
SS08
SS10
SS12
SS13
SS28
SS29
SS02
SS05
SS07
SS09
SS14
SS17
SS18
SS19
SS20
SS21
SS22
SS25
SS26
SS27
SS31
CR+PR
PD
Pre-normalization
Post-normalization

**b**

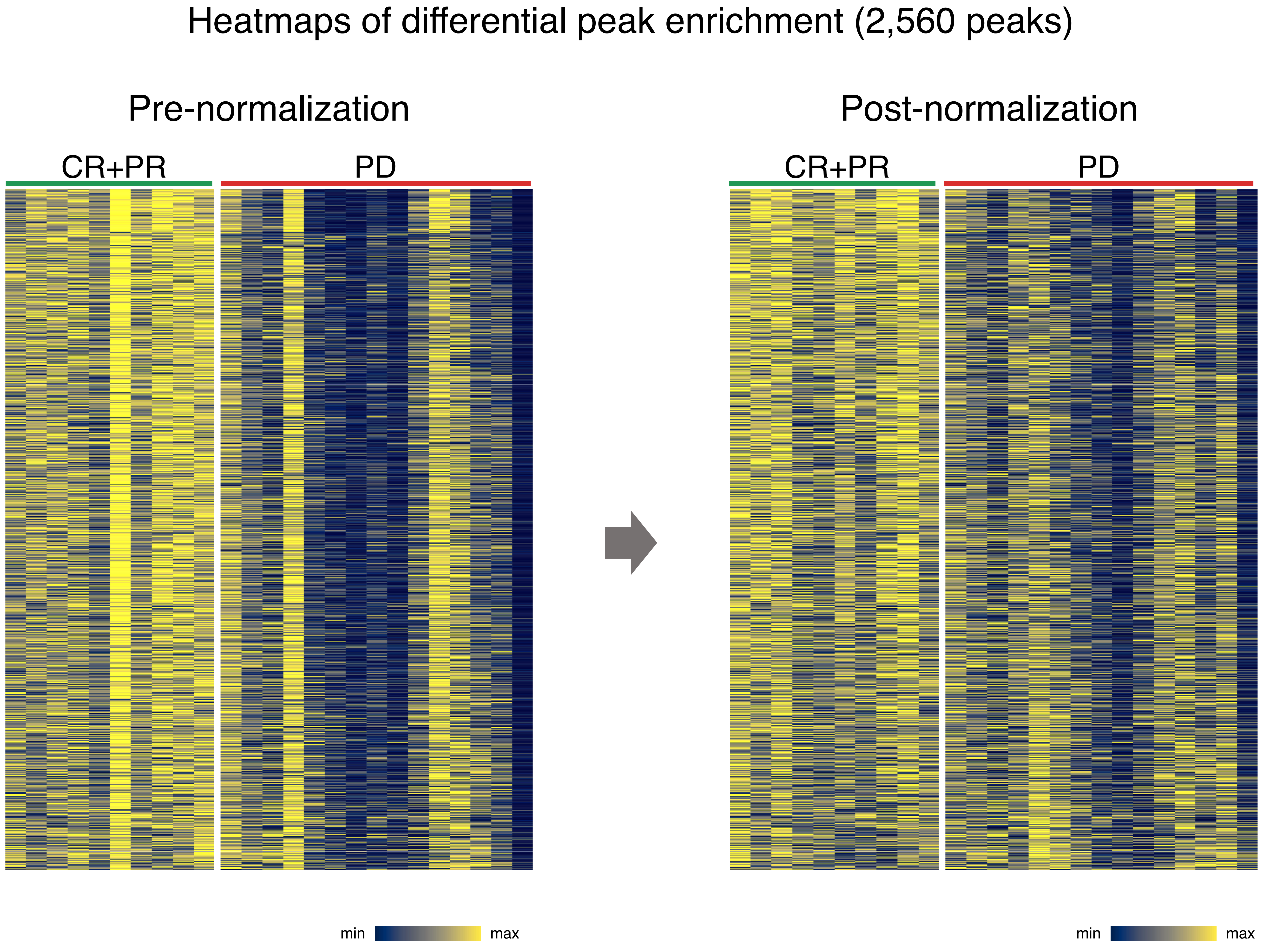
Heatmaps of differential peak enrichment (2,560 peaks)
Pre-normalization
Post-normalization
CR+PR
PD
min
max

**Supplementary Fig. 8.** Target features. Nine targets selected using ATAC-seq analysis are shown at the corresponding chromosome regions. An integrated ATAC-seq genome browser showed up to four types of data. Reference University of California, Santa Cruz (UCSC) genes are shown at the designated scale. Histone H3K27ac (acetyl H3K27), identified by ChIP-seq from seven cell lines (ENCODE) discriminated active enhancer marks; DNase hypersensitivity clusters from 125 cell types (ENCODE V3) represent open chromatin regions. This genomic information defines the regulatory elements of the respective sites. Transcription factor ChIP-seq clusters with 161 factors (ENCODE with factorbook motifs) are displayed at the indicated genomic regions. Shaded boxes indicate genomic regions of the nine targets.

Supplementary Fig. 8

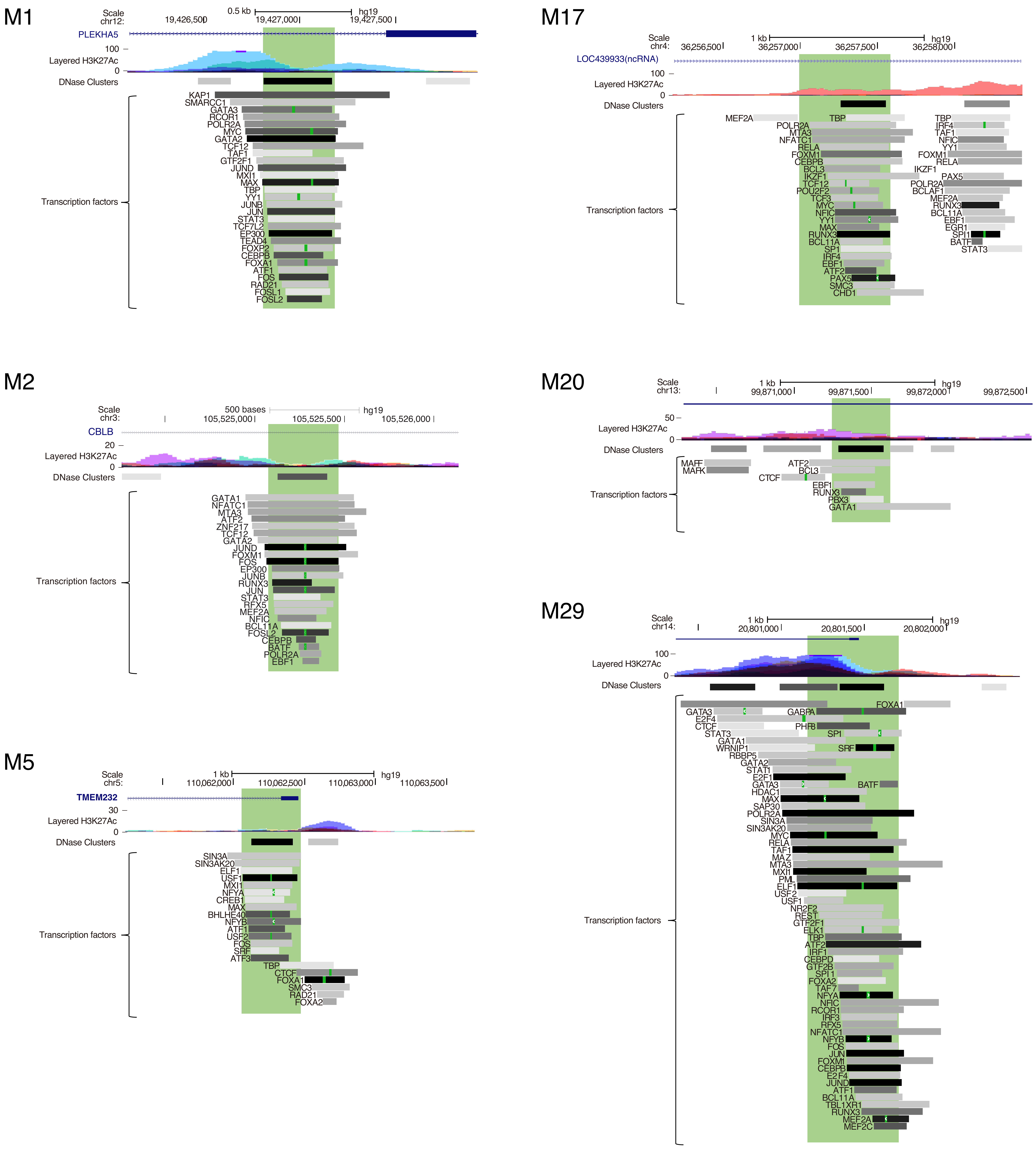

# Supplementary Fig. 8

## M30

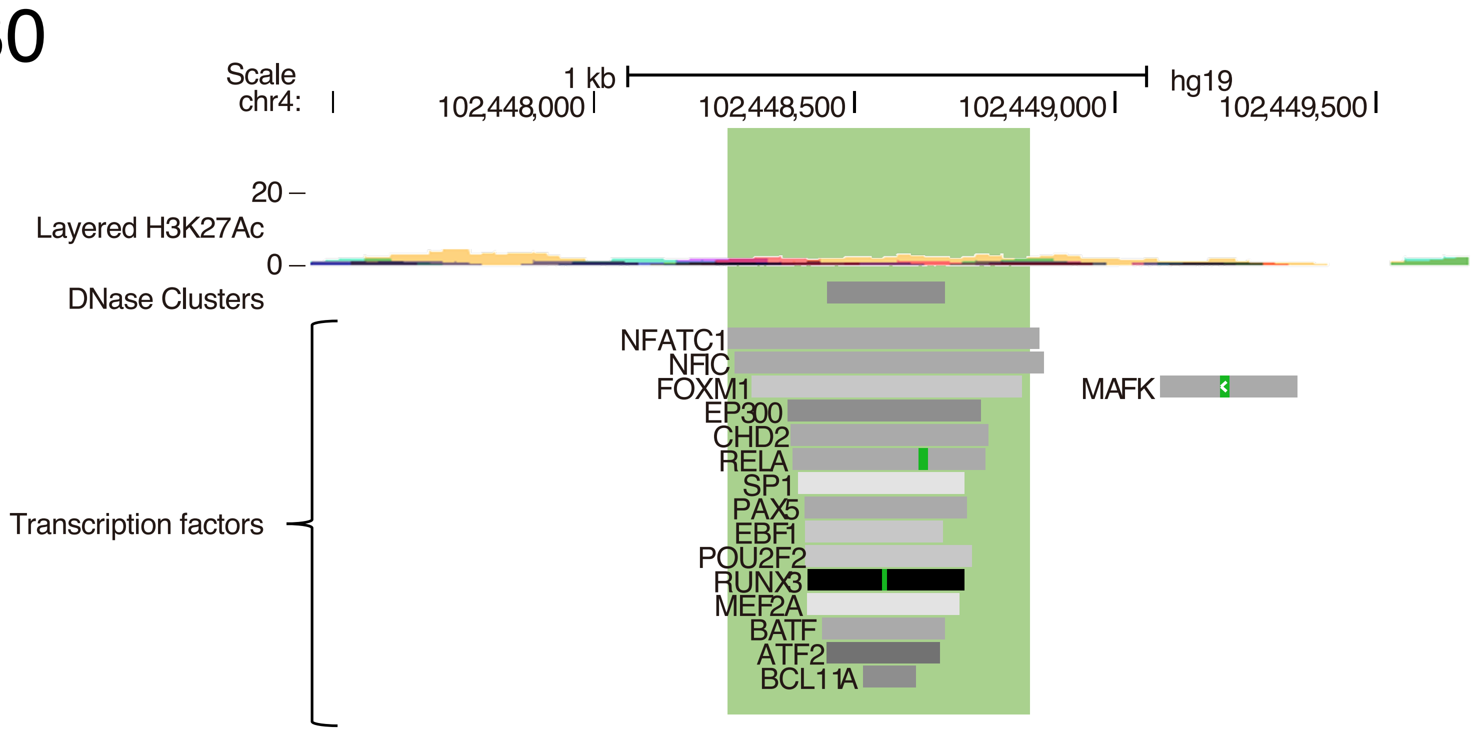


## M35

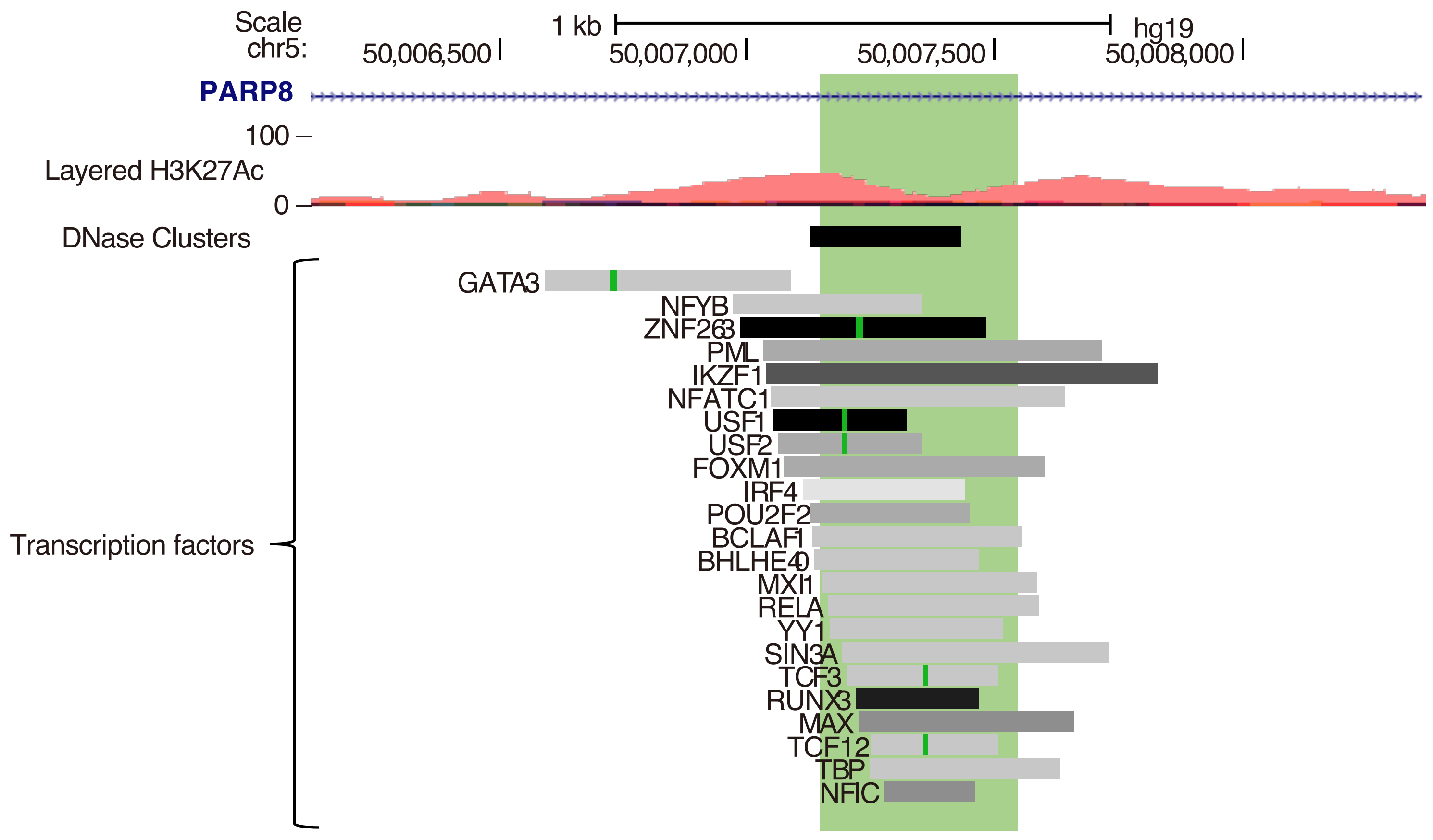


## M36

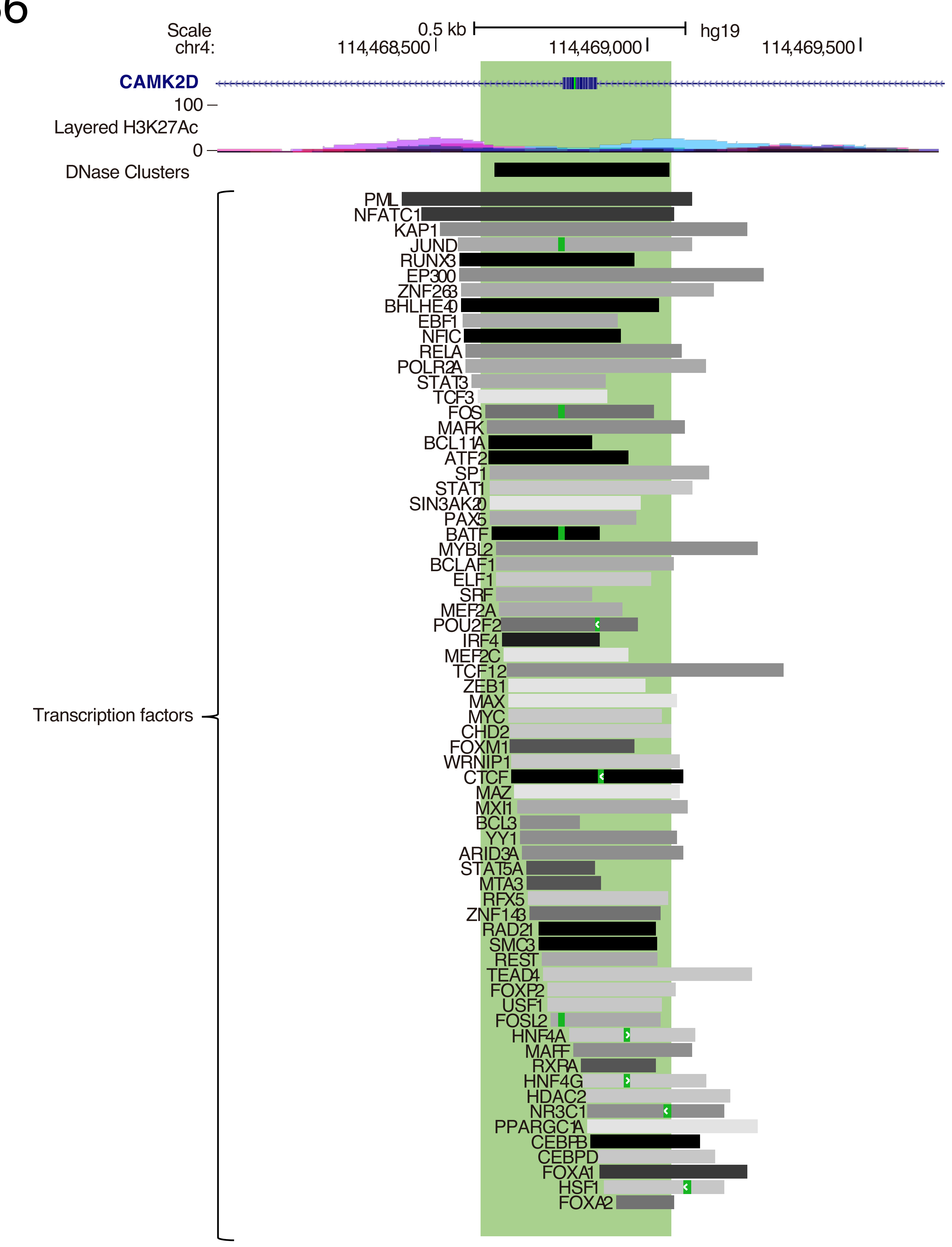

**Supplementary Fig. 9.** Evaluation of targets as markers for prediction of clinical outcomes in the discovery cohort. For the responder group (R, CR+PR) and non-responder group (NR, PD+SD), targets are displayed with normalized area values (left plots). Horizontal bar indicates the mean. Normalized area values of the responder group (CR+PR) and progressive disease group (PD) minus the threshold (Supplementary Table 6) are presented in descending order on the horizontal axes of waterfall graphs (middle graphs). Threshold values were used to determine clinical outcomes of anti-PD-1 therapy using progression-free survival (PFS) curves (right graphs). Tumor progression scores for patients above and below the threshold are shown in blue and red, respectively. Median PFS (mPFS) time was calculated using Kaplan–Meier survival analysis. Survival curves of the two groups were compared using the two-sided log-rank (Mantel–Cox) test in a single comparison. *n.r.*, not reached.

Supplementary Fig. 9

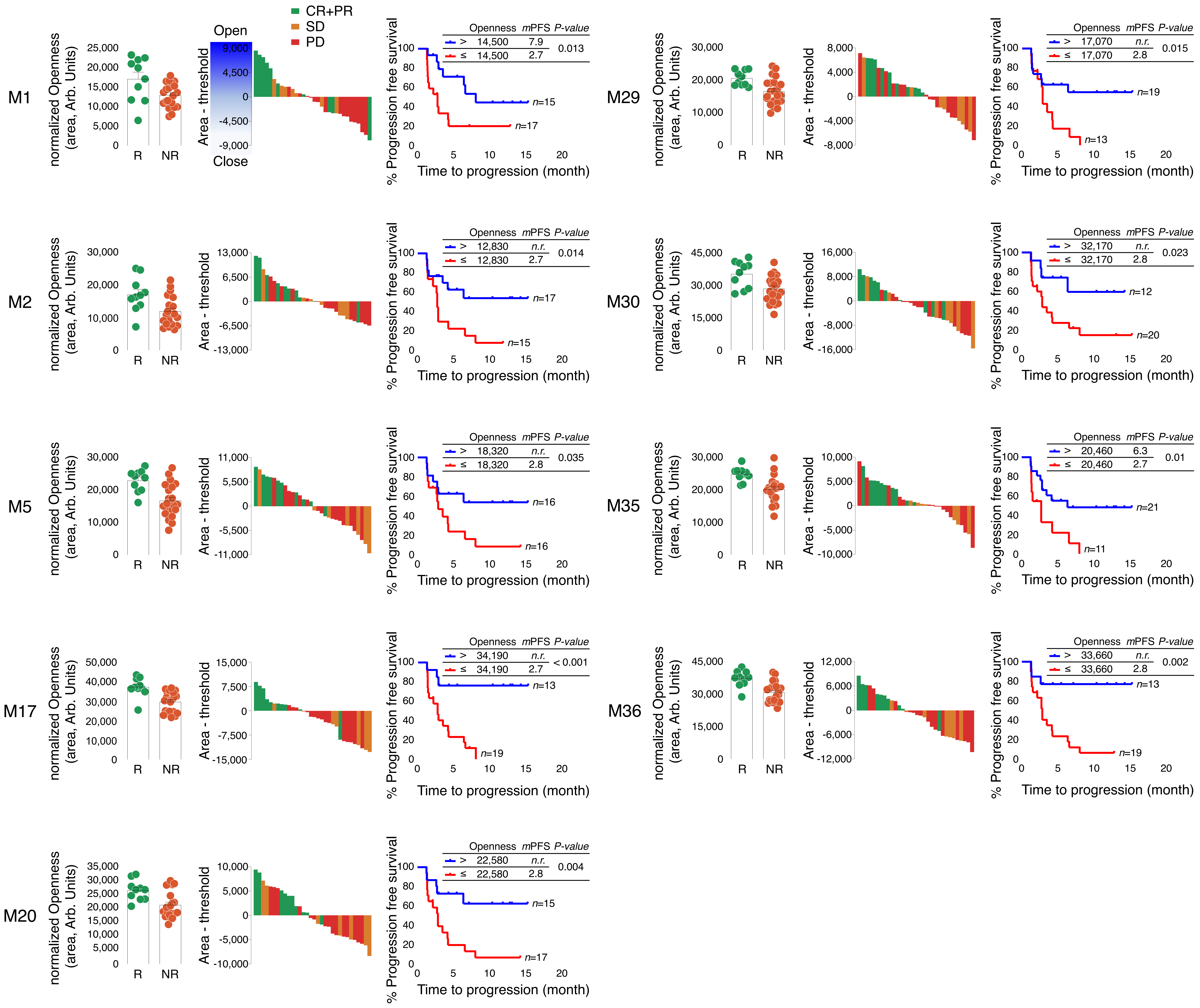

**Supplementary Fig. 10.** Evaluation of targets as markers for prediction of clinical outcomes in the validation cohort. Targets for the responder group (R, CR+PR) and non-responder group (NR, PD+SD) are shown with normalized area values (left plots). Horizontal bar indicates the mean area of each group. Normalized area values of the responder group (CR+PR) and progressive disease group (PD) minus threshold (Supplementary Table 8) were plotted in descending order on the horizontal axis on a waterfall graph (middle graphs). Threshold values were used to determine the clinical outcomes of anti-PD-1 therapy using a PFS curve (right graphs). Tumor progression scores of patients above and below the threshold are shown in blue and red, respectively. Median PFS (mPFS) time was calculated using Kaplan–Meier survival analysis. Survival curves of the two groups were compared using the two-sided log-rank (Mantel–Cox) test in a single comparison. *n.r.*, not reached.

Supplementary Fig. 10

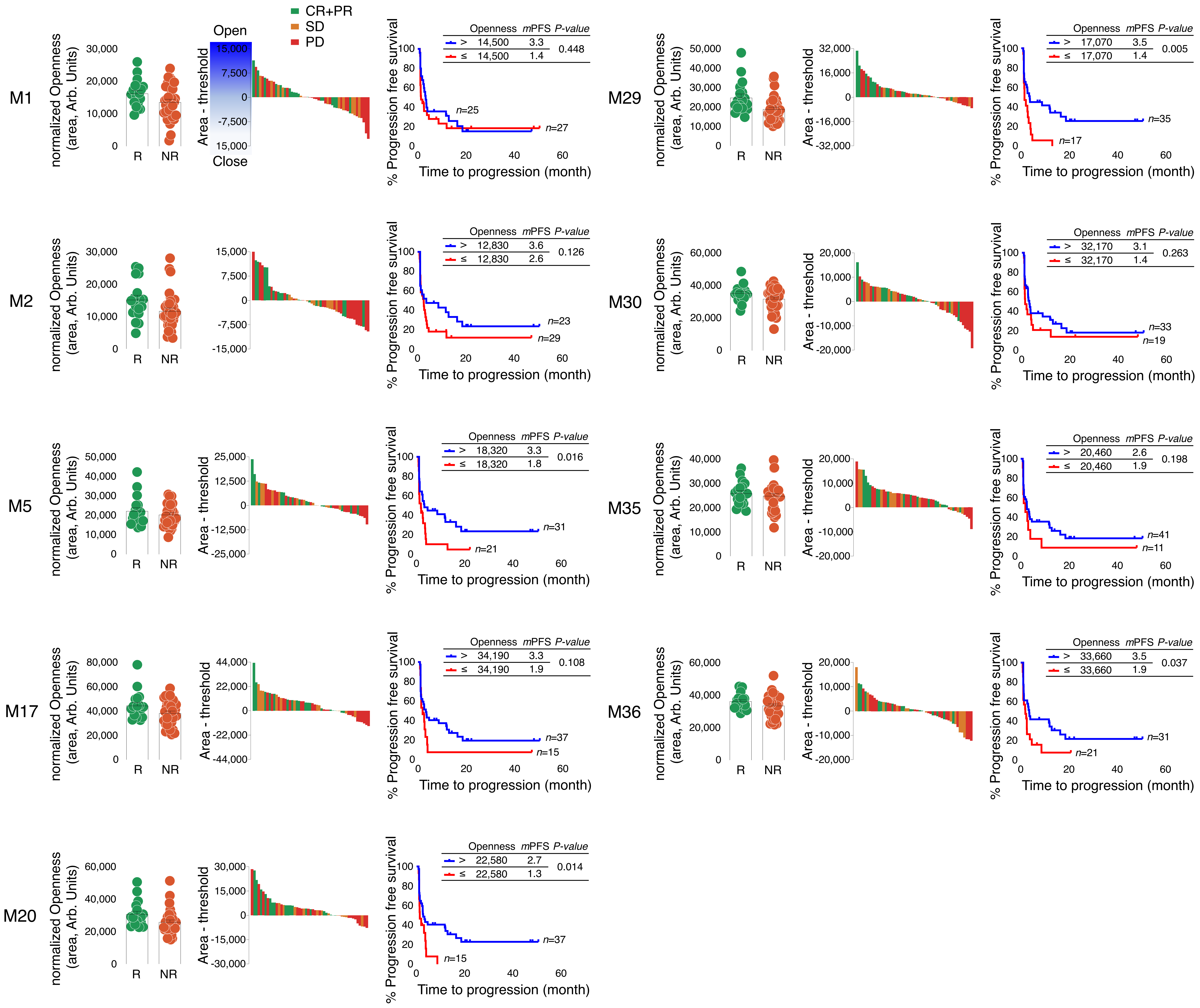

**Supplementary Table 1.** Clinical characteristics of cohort 1 patients with gastric cancer (GC), used as a discovery dataset

| Sample ID | MSS/EBV/PD-L1 status |
|---|---|
| SS01 | MSI-H/(-)/(+) |
| SS11 | MSI-H/(-)/(+) |
| SS04 | MSI-H/(-)/(+) |
| SS06 | MSS/(+)/(+) |
| SS08 | MSS/(+)/(+) |
| SS10 | MSS/(+)/(+) |
| SS12 | MSS/(-)/n.a. |
| SS13 | MSS/(-)/(-) |
| SS28 | MSS/(+)/(+) |
| SS29 | MSI-H/(-)/(+) |
| SS03 | MSS/(-)/(-) |
| SS15 | MSS/(-)/(+) |
| SS16 | MSS/(-)/(+) |
| SS23 | MSS/(-)/(-) |
| SS24 | MSS/(-)/(+) |
| SS30 | MSS/(-)/(+) |
| SS32 | MSS/(-)/(-) |
| SS02 | MSS/(-)/(+) |
| SS05 | MSS/(-)/n.a. |
| SS07 | MSS/(-)/n.a. |
| SS09 | MSS/(-)/(-) |
| SS14 | MSS/(-)/(+) |
| SS17 | MSS/(-)/(+) |
| SS18 | MSS/(-)/(-) |
| SS19 | MSS/(-)/(+) |
| SS20 | MSI-H/(-)/(+) |
| SS21 | MSS/(-)/(+) |
| SS22 | MSS/(-)/(-) |
| SS25 | MSS/(-)/n.a. |
| SS26 | MSS/(-)/(+) |
| SS27 | MSS/(-)/(+) |
| SS31 | MSS/(-)/(-) |

MSS, microsatellite stable; MSI-H, microsatellite instability high; EBV, Ebstein Barr virus; PD-L1, programmed death ligand-1; n.a., not available

**Supplementary Table 2.** Clinical characteristics of cohort 2 patients with GC, used as a validation dataset

| Sample ID | MSS/EBV/PD-L1 status |
|---|---|
| SS005 | MSI-H/(-)/(+) |
| SS023 | MSS/(-)/(+) |
| SS028 | MSI-H/n.a./(+) |
| SS004 | MSI-H/(-)/(+) |
| SS006 | MSS/(+)/(+) |
| SS009 | MSS/(-)/(+) |
| SS035 | MSS/(-)/(+) |
| SS038 | MSS(-)/(-) |
| SS042 | MSS/(-)/(-) |
| SS043 | MSS/(-)/(+) |
| SS046 | MSI-H/(-)/(+) |
| SS047 | MSS/(-)/(+) |
| SS048 | MSS/(-)/(+) |
| SS052 | MSS/(-)/(+) |
| SS053 | MSS/(-)/(+) |
| SS058 | MSS/(-)/(+) |
| SS059 | MSS/(-)/(+) |
| SS065 | MSS/(-)/(+) |
| SS007 | MSS/(+)/(-) |
| SS011 | MSS/(-)/n.a. |
| SS014 | MSS/(-)/(+) |
| SS016 | MSS/(-)/(-) |
| SS017 | MSS/(-)/(-) |
| SS019 | MSI-H/(-)/(+) |
| SS033 | MSS/(-)/(-) |
| SS049 | MSS/(-)/(-) |
| SS054 | MSI-H/(-)/(+) |
| SS055 | MSS/(-)/(-) |
| SS060 | MSS/(-)/(-) |
| SS061 | MSS/(+)/(+) |
| SS001 | MSS/(-)/(-) |
| SS002 | MSS/(-)/(-) |

| | |
|---|---|
| SS003 | MSS/(-)/(-) |
| SS010 | MSS/(-)/n.a. |
| SS012 | MSS/(-)/(-) |
| SS013 | MSS/(-)/n.a. |
| SS018 | MSS/(-)/(-) |
| SS034 | MSS/(+)/(+) |
| SS036 | MSS/(-)/(-) |
| SS037 | MSS/(-)/(+) |
| SS039 | MSS/(-)/(+) |
| SS041 | MSS/(-)/(-) |
| SS044 | MSS/(-)/(+) |
| SS045 | MSS/(-)/(-) |
| SS050 | MSS/(-)/(+) |
| SS051 | MSS/(-)/(-) |
| SS056 | MSS/(-)/(-) |
| SS057 | MSS/(+)/(+) |
| SS062 | MSS/(-)/(+) |
| SS063 | MSS/(-)/(+) |
| SS064 | MSS/(-)/(+) |
| SS066 | MSS/(+)/(+) |

MSS, microsatellite stable; MSI-H, microsatellite instability high; EBV, Ebstein Barr virus; PD-L1, programmed death ligand-1; n.a., not available

**Supplementary Table 3.** Assay for transposase-accessible chromatin using sequencing (ATAC-seq) metadata and quality control statistics

| Sample ID | Total reads | Aligned reads | % Aligned | % Duplicated | % Mitochondria | NRF | PBC | FRiP | TSS enrichment |
|---|---|---|---|---|---|---|---|---|---|
| SS01 | 79,369,131 | 72,958,765 | 91.9% | 51.3% | 31.4% | 0.98 | 0.86 | 0.60 | 16.4 |
| SS02 | 87,638,376 | 80,039,100 | 91.3% | 43.0 % | 21.4 % | 0.98 | 0.83 | 0.47 | 16.0 |
| SS03 | 126,685,260 | 115,837,870 | 91.4% | 45.6% | 21.2% | 0.98 | 0.80 | 0.55 | 26.8 |
| SS04 | 66,576,763 | 60,989,804 | 91.6% | 43.1% | 26.0% | 0.98 | 0.89 | 0.64 | 28.2 |
| SS05 | 69,794,733 | 63,072,918 | 90.4% | 46.4% | 26.2% | 0.98 | 0.85 | 0.54 | 18.1 |
| SS06 | 110,916,650 | 102,146,159 | 92.1% | 46.1% | 26.9% | 0.98 | 0.86 | 0.53 | 11.9 |
| SS07 | 87,534,113 | 80,315,836 | 91.8% | 50.5% | 29.0% | 0.98 | 0.83 | 0.50 | 14.2 |
| SS08 | 79,366,311 | 73,459,580 | 92.6% | 41.6% | 23.9% | 0.98 | 0.88 | 0.53 | 15.7 |
| SS09 | 111,528,381 | 102,440,329 | 91.9% | 47.7% | 21.2% | 0.98 | 0.77 | 0.51 | 16.9 |
| SS10 | 106,022,192 | 97,579,178 | 92.0% | 46.6% | 24.4% | 0.98 | 0.83 | 0.51 | 21.3 |
| SS11 | 71,755,642 | 66,493,486 | 92.7% | 38.3% | 20.8% | 0.98 | 0.88 | 0.46 | 16.3 |
| SS12 | 88,065,358 | 82,845,759 | 94.1% | 47.2% | 24.7% | 0.98 | 0.82 | 0.42 | 14.3 |
| SS13 | 64,060,244 | 60,505,431 | 94.5% | 44.3% | 21.5% | 0.97 | 0.83 | 0.44 | 19.2 |
| SS14 | 103,257,981 | 97,746,391 | 94.7% | 39.3% | 13.3% | 0.98 | 0.76 | 0.51 | 16.8 |
| SS15 | 124,158,216 | 116,490,224 | 93.8% | 53.5% | 16.8% | 0.97 | 0.66 | 0.57 | 16.3 |
| SS16 | 73,535,141 | 69,458,094 | 94.5% | 36.6% | 18.5% | 0.98 | 0.87 | 0.53 | 21.9 |
| SS17 | 84,446,831 | 79,526,537 | 94.2% | 38.0% | 14.7% | 0.98 | 0.80 | 0.38 | 16.3 |
| SS18 | 61,138,757 | 58,835,255 | 96.2% | 36.3% | 18.9% | 0.98 | 0.87 | 0.49 | 26.6 |
| SS19 | 74,499,318 | 71,572,361 | 96.1% | 44.2% | 21.4% | 0.98 | 0.81 | 0.41 | 26.2 |
| SS20 | 80,080,760 | 76,640,704 | 95.7% | 41.4% | 17.6% | 0.98 | 0.80 | 0.48 | 16.1 |
| SS21 | 70,826,527 | 67,917,053 | 95.9% | 40.5% | 18.0% | 0.98 | 0.82 | 0.54 | 17.2 |
| SS22 | 89,838,238 | 86,206,816 | 96.0% | 45.4% | 22.9% | 0.97 | 0.83 | 0.48 | 14.0 |
| SS23 | 75,306,813 | 69,825,407 | 92.7% | 37.3% | 18.0% | 0.98 | 0.86 | 0.35 | 10.8 |
| SS24 | 115,656,817 | 107,321,051 | 92.8% | 39.1% | 14.7% | 0.98 | 0.79 | 0.37 | 12.3 |
| SS25 | 89,273,685 | 82,918,784 | 92.9% | 49.1% | 24.5% | 0.98 | 0.79 | 0.42 | 15.3 |
| SS26 | 107,117,518 | 99,277,606 | 92.7% | 32.6% | 11.6% | 0.98 | 0.84 | 0.48 | 13.4 |
| SS27 | 52,636,072 | 48,671,987 | 92.5% | 38.5% | 20.4% | 0.98 | 0.87 | 0.44 | 15.8 |
| SS28 | 64,582,832 | 60,171,173 | 93.2% | 43.7% | 23.8% | 0.98 | 0.86 | 0.42 | 14.8 |

| | | | | | | | | | |
|---|---|---|---|---|---|---|---|---|---|
| SS29 | 80,226,651 | 75,580,819 | 94.2% | 57.0% | 33.6% | 0.97 | 0.81 | 0.46 | 23.0 |
| SS30 | 101,999,249 | 95,839,289 | 94.0% | 54.0% | 30.3% | 0.97 | 0.81 | 0.39 | 13.5 |
| SS31 | 49,007,843 | 45,924,143 | 93.7% | 30.1% | 14.7% | 0.98 | 0.89 | 0.61 | 24.0 |
| SS32 | 114,145,932 | 107,358,491 | 94.1% | 53.5% | 24.7% | 0.97 | 0.74 | 0.54 | 20.5 |

NRF, non-redundant fraction: number of distinct uniquely mapping reads after removal of duplicates / total number of reads. The value greater than 0.9 is preferred.

PBC, polymerase chain reaction (PCR) bottlenecking coefficient: PBC = M/M_DISTINCT, where M is the number of genomic locations to which exactly one read maps uniquely and M_DISTINCT is the number of distinct genomic locations to which some reads map uniquely. The value greater than 0.9 is preferred.

FRiP, fraction of reads in peaks: The number of mapped reads that overlap the peak was divided by the total number of mapped reads. FRiP score greater than 0.3 is preferred, though the value greater than 0.2 is acceptable.

Transcription Start Site (TSS) enrichment: The maximum ratio calculated as average aligned reads for every 51 bp in the TSS was divided by the average aligned reads for each single base at the TSS boundary (± 1,900 to 2,000 bp from TSS). In *hg19* Refseq TSS annotation, TSS enrichment score greater than 10 is ideal, though the value greater than 6 is acceptable.

**Supplementary Table 4.** ATAC-seq signal detection using three peak-caller software suites

| | HOMER suite | MACS2 | Cisgenome |
|---|---|---|---|
| Statistical method | Poisson | Poisson | Negative binomial |
| Significance measure | *P*-value | loglikelihood | Fold change |
| Parameters | $P < 0.01$ | nolambda | Read extension length 150 bp |
| | Style factor | nomodel | Bin size 50 bp |
| | tbp 3 (maximum tags per bp) | shift 100 | Maximum gap 50 bp |
| | Region (variable peak width) | extsize 200 | Minimum peak length 100 bp |
| Filtering out | Excluding chrM, chrY and unassigned chr (chrUn) | | |
| Peak calling | Merging peaks of individual samples | Multiple group comparison | Multiple group comparison |
| Peaks | Total 85,625 | Differential 161,838 | Differential 20,969 |
| Common peaks among all three suites | 2,560 | | |

**Supplementary Table 5.** Normalization factors with –5, –20, and –50 ranked controls

| | | Normalization factor (*F*) | | |
|---|---|---|---|---|
| Response | Sample ID | Ranked –5 | Ranked –20 | Ranked –50 |
| Responders (CR+PR) | SS01 | 1.11 | 1.14 | 1.16 |
| | SS11 | 1.05 | 1.07 | 1.09 |
| | SS04 | 1.07 | 1.11 | 1.14 |
| | SS06 | 0.95 | 0.96 | 0.98 |
| | SS08 | 1.01 | 1.06 | 1.06 |
| | SS10 | 0.75 | 0.75 | 0.75 |
| | SS12 | 0.92 | 0.97 | 0.97 |
| | SS13 | 0.90 | 0.92 | 0.91 |
| | SS28 | 0.97 | 1.02 | 1.04 |
| | SS29 | 0.90 | 0.91 | 0.93 |
| Non-responders (SD) | SS03 | 0.78 | 0.76 | 0.77 |
| | SS15 | 0.74 | 0.74 | 0.72 |
| | SS16 | 1.11 | 1.15 | 1.16 |
| | SS23 | 1.20 | 1.11 | 1.12 |
| | SS24 | 1.24 | 1.18 | 1.17 |
| | SS30 | 0.87 | 0.78 | 0.76 |
| | SS32 | 0.82 | 0.74 | 0.72 |
| Non-responders (PD) | SS02 | 0.90 | 0.94 | 0.95 |
| | SS05 | 1.03 | 1.06 | 1.08 |
| | SS07 | 1.07 | 1.04 | 1.02 |
| | SS09 | 0.82 | 0.82 | 0.82 |
| | SS14 | 1.39 | 1.38 | 1.37 |
| | SS17 | 1.44 | 1.36 | 1.33 |
| | SS18 | 1.29 | 1.36 | 1.41 |
| | SS19 | 1.05 | 1.10 | 1.10 |
| | SS20 | 1.13 | 1.12 | 1.11 |
| | SS21 | 0.93 | 0.97 | 0.96 |
| | SS22 | 0.82 | 0.84 | 0.83 |
| | SS25 | 0.97 | 0.99 | 1.02 |
| | SS26 | 1.15 | 1.04 | 1.02 |
| | SS27 | 1.27 | 1.25 | 1.24 |
| | SS31 | 1.44 | 1.42 | 1.43 |

**Supplementary Table 6.** Statistical evaluation of targets for clinical outcome prediction in the discovery cohort.

| Target ID | AUROC | Thr. | R | NR | Sensitivity | Specificity | Target ID | AUROC | Thr. | R | NR | Sensitivity | Specificity |
|---|---|---|---|---|---|---|---|---|---|---|---|---|---|
| M1 | 0.745 | > | 7 | 8 | 70.0% | 63.6% | M35 | 0.873 | > | 10 | 11 | 100.0% | 50.0% |
| | | ≤ | 3 | 14 | | | | | ≤ | 0 | 11 | | |
| M2 | 0.777 | > | 9 | 8 | 90.0% | 63.6% | M36 | 0.868 | > | 9 | 4 | 90.0% | 81.8% |
| | | ≤ | 1 | 14 | | | | | ≤ | 1 | 18 | | |
| M3 | 0.886 | > | 9 | 4 | 90.0% | 81.8% | M37 | 0.750 | > | 8 | 5 | 80.0% | 77.3% |
| | | ≤ | 1 | 18 | | | | | ≤ | 2 | 17 | | |
| M4 | 0.718 | > | 7 | 7 | 70.0% | 68.2% | M38 | 0.805 | > | 7 | 6 | 70.0% | 72.7% |
| | | ≤ | 3 | 15 | | | | | ≤ | 3 | 16 | | |
| M5 | 0.841 | > | 9 | 7 | 90.0% | 68.2% | M39 | 0.755 | > | 9 | 7 | 90.0% | 68.2% |
| | | ≤ | 1 | 15 | | | | | ≤ | 1 | 15 | | |
| M6 | 0.768 | > | 7 | 5 | 70.0% | 77.3% | M40 | 0.823 | > | 9 | 5 | 90.0% | 77.3% |
| | | ≤ | 3 | 17 | | | | | ≤ | 1 | 17 | | |
| M7 | 0.845 | > | 9 | 7 | 90.0% | 68.2% | M41 | 0.745 | > | 8 | 8 | 80.0% | 63.6% |
| | | ≤ | 1 | 15 | | | | | ≤ | 2 | 14 | | |
| M8 | 0.759 | > | 8 | 4 | 80.0% | 81.8% | M42 | 0.764 | > | 7 | 5 | 70.0% | 77.3% |
| | | ≤ | 2 | 18 | | | | | ≤ | 3 | 17 | | |
| M9 | 0.761 | > | 8 | 13 | 80.0% | 40.9% | M43 | 0.736 | > | 7 | 6 | 70.0% | 72.7% |
| | | ≤ | 2 | 9 | | | | | ≤ | 3 | 16 | | |
| M10 | 0.718 | > | 9 | 10 | 90.0% | 54.5% | M44 | 0.727 | > | 7 | 4 | 70.0% | 81.8% |
| | | ≤ | 1 | 12 | | | | | ≤ | 3 | 18 | | |
| M11 | 0.786 | > | 9 | 11 | 90.0% | 50.0% | M45 | 0.705 | > | 7 | 6 | 70.0% | 72.7% |
| | | ≤ | 1 | 11 | | | | | ≤ | 3 | 16 | | |
| M12 | 0.705 | > | 9 | 12 | 90.0% | 45.5% | M46 | 0.682 | > | 7 | 9 | 70.0% | 59.1% |
| | | ≤ | 1 | 10 | | | | | ≤ | 3 | 13 | | |
| M13 | 0.809 | > | 8 | 5 | 80.0% | 77.3% | M47 | 0.805 | > | 9 | 7 | 90.0% | 68.2% |
| | | ≤ | 2 | 17 | | | | | ≤ | 1 | 15 | | |
| M14 | 0.759 | > | 9 | 8 | 90.0% | 63.6% | M48 | 0.814 | > | 7 | 3 | 70.0% | 86.4% |
| | | ≤ | 1 | 14 | | | | | ≤ | 3 | 19 | | |
| M15 | 0.723 | > | 7 | 9 | 70.0% | 59.1% | M49 | 0.689 | > | 8 | 8 | 80.0% | 63.6% |
| | | ≤ | 3 | 13 | | | | | ≤ | 2 | 14 | | |
| M16 | 0.750 | > | 9 | 8 | 90.0% | 63.6% | M50 | 0.791 | > | 9 | 9 | 90.0% | 59.1% |
| | | ≤ | 1 | 14 | | | | | ≤ | 1 | 13 | | |
| M17 | 0.905 | > | 9 | 4 | 90.0% | 81.8% | M51 | 0.786 | > | 9 | 7 | 90.0% | 68.2% |
| | | ≤ | 1 | 18 | | | | | ≤ | 1 | 15 | | |
| M18 | 0.814 | > | 9 | 8 | 90.0% | 63.6% | M52 | 0.768 | > | 9 | 9 | 90.0% | 59.1% |
| | | ≤ | 1 | 14 | | | | | ≤ | 1 | 13 | | |
| M19 | 0.814 | > | 10 | 7 | 100.0% | 68.2% | M53 | 0.755 | > | 9 | 9 | 90.0% | 59.1% |
| | | ≤ | 0 | 15 | | | | | ≤ | 1 | 13 | | |
| M20 | 0.791 | > | 9 | 6 | 90.0% | 72.7% | M54 | 0.700 | > | 7 | 6 | 70.0% | 72.7% |
| | | ≤ | 1 | 16 | | | | | ≤ | 3 | 16 | | |
| M21 | 0.818 | > | 9 | 6 | 90.0% | 72.7% | M55 | 0.759 | > | 8 | 7 | 80.0% | 68.2% |
| | | ≤ | 1 | 16 | | | | | ≤ | 2 | 15 | | |
| M22 | 0.727 | > | 8 | 9 | 80.0% | 59.1% | M56 | 0.814 | > | 9 | 7 | 90.0% | 68.2% |
| | | ≤ | 2 | 13 | | | | | ≤ | 1 | 15 | | |
| M23 | 0.832 | > | 8 | 5 | 80.0% | 77.3% | M57 | 0.877 | > | 9 | 4 | 90.0% | 81.8% |
| | | ≤ | 2 | 17 | | | | | ≤ | 1 | 18 | | |

| | | | | | | | | | | | | | |
|---|---|---|---|---|---|---|---|---|---|---|---|---|---|
| M24 | 0.736 | > | 7 | 7 | 70.0% | 68.2% | M58 | 0.764 | > | 7 | 5 | 70.0% | 77.3% |
| | | ≤ | 3 | 15 | | | | | ≤ | 3 | 17 | | |
| M25 | 0.741 | > | 8 | 8 | 80.0% | 63.6% | M59 | 0.764 | > | 7 | 4 | 70.0% | 81.8% |
| | | ≤ | 2 | 14 | | | | | ≤ | 3 | 18 | | |
| M26 | 0.755 | > | 9 | 9 | 90.0% | 59.1% | M60 | 0.832 | > | 8 | 4 | 80.0% | 81.8% |
| | | ≤ | 1 | 13 | | | | | ≤ | 2 | 18 | | |
| M27 | 0.818 | > | 7 | 5 | 70.0% | 77.3% | M61 | 0.718 | > | 8 | 7 | 80.0% | 68.2% |
| | | ≤ | 3 | 17 | | | | | ≤ | 2 | 15 | | |
| M28 | 0.823 | > | 9 | 8 | 90.0% | 63.6% | M62 | 0.777 | > | 9 | 7 | 90.0% | 68.2% |
| | | ≤ | 1 | 14 | | | | | ≤ | 1 | 15 | | |
| M29 | 0.777 | > | 10 | 9 | 100.0% | 59.1% | M63 | 0.773 | > | 9 | 8 | 90.0% | 63.6% |
| | | ≤ | 0 | 13 | | | | | ≤ | 1 | 14 | | |
| M30 | 0.786 | > | 7 | 5 | 70.0% | 77.3% | M64 | 0.800 | > | 9 | 6 | 90.0% | 72.7% |
| | | ≤ | 3 | 17 | | | | | ≤ | 1 | 16 | | |
| M31 | 0.755 | > | 8 | 8 | 80.0% | 63.6% | M65 | 0.859 | > | 8 | 3 | 80.0% | 86.4% |
| | | ≤ | 2 | 14 | | | | | ≤ | 2 | 19 | | |
| M32 | 0.791 | > | 9 | 9 | 90.0% | 59.1% | M66 | 0.777 | > | 7 | 5 | 70.0% | 77.3% |
| | | ≤ | 1 | 13 | | | | | ≤ | 3 | 17 | | |
| M33 | 0.818 | > | 9 | 9 | 90.0% | 59.1% | M67 | 0.764 | > | 8 | 6 | 80.0% | 72.7% |
| | | ≤ | 1 | 13 | | | | | ≤ | 2 | 16 | | |
| M34 | 0.764 | > | 9 | 8 | 90.0% | 63.6% | | | | | | | |
| | | ≤ | 1 | 14 | | | | | | | | | |

**Supplementary Table 7.** The accuracy of each target was calculated based on the proportion of true positives and true negatives in all evaluated samples.

| Target ID | ACC** |
|---|---|
| M36 | 84.4% |
| M17 | 84.4% |
| M20 | 78.1% |
| M30 | 75.0% |
| M5 | 75.0% |
| M29 | 71.9% |
| M2 | 71.9% |
| M1 | 65.6% |
| M35 | 65.6% |

ACC**: Accuracy = (TP + TN) / (TP + FP + TN + FN)
TP = True positive; FP = False positive
TN = True negative; FN = False negative

**Supplementary Table 8.** Statistical evaluation of targets for clinical outcome prediction in the validation cohort.

| Target ID | AUROC | Thr. | R | NR | Sensitivity | Specificity | Target ID | AUROC | Thr. | R | NR | Sensitivity | Specificity |
|---|---|---|---|---|---|---|---|---|---|---|---|---|---|
| M1 | 0.648 | > | 12 | 13 | 66.7% | 61.8% | M35 | 0.544 | > | 16 | 25 | 88.9% | 26.5% |
| | | ≤ | 6 | 21 | | | | | ≤ | 2 | 9 | | |
| M2 | 0.703 | > | 12 | 11 | 66.7% | 67.6% | M36 | 0.619 | > | 14 | 17 | 77.8% | 50.0% |
| | | ≤ | 6 | 23 | | | | | ≤ | 4 | 17 | | |
| M3 | 0.611 | > | 16 | 22 | 88.9% | 35.3% | M37 | 0.660 | > | 12 | 16 | 66.7% | 52.9% |
| | | ≤ | 2 | 12 | | | | | ≤ | 6 | 18 | | |
| M4 | 0.660 | > | 16 | 20 | 88.9% | 41.2% | M38 | 0.592 | > | 14 | 19 | 77.8% | 44.1% |
| | | ≤ | 2 | 14 | | | | | ≤ | 4 | 15 | | |
| M5 | 0.560 | > | 12 | 19 | 66.7% | 44.1% | M39 | 0.596 | > | 16 | 22 | 88.9% | 35.3% |
| | | ≤ | 6 | 15 | | | | | ≤ | 2 | 12 | | |
| M6 | 0.389 | > | 7 | 19 | 38.9% | 44.1% | M40 | 0.637 | > | 14 | 22 | 77.8% | 35.3% |
| | | ≤ | 11 | 15 | | | | | ≤ | 4 | 12 | | |
| M7 | 0.426 | > | 9 | 21 | 50.0% | 38.2% | M41 | 0.508 | > | 13 | 25 | 72.2% | 26.5% |
| | | ≤ | 9 | 13 | | | | | ≤ | 5 | 9 | | |
| M8 | 0.467 | > | 8 | 18 | 44.4% | 47.1% | M42 | 0.616 | > | 8 | 13 | 44.4% | 61.8% |
| | | ≤ | 10 | 16 | | | | | ≤ | 10 | 21 | | |
| M9 | 0.699 | > | 15 | 15 | 83.3% | 55.9% | M43 | 0.617 | > | 13 | 17 | 72.2% | 50.0% |
| | | ≤ | 3 | 19 | | | | | ≤ | 5 | 17 | | |
| M10 | 0.529 | > | 15 | 20 | 83.3% | 41.2% | M44 | 0.588 | > | 8 | 14 | 44.4% | 58.8% |
| | | ≤ | 3 | 14 | | | | | ≤ | 10 | 20 | | |
| M11 | 0.694 | > | 14 | 19 | 77.8% | 44.1% | M45 | 0.636 | > | 15 | 21 | 83.3% | 38.2% |
| | | ≤ | 4 | 15 | | | | | ≤ | 3 | 13 | | |
| M12 | 0.709 | > | 12 | 13 | 66.7% | 61.8% | M46 | 0.605 | > | 11 | 15 | 61.1% | 55.9% |
| | | ≤ | 6 | 21 | | | | | ≤ | 7 | 19 | | |
| M13 | 0.645 | > | 13 | 20 | 72.2% | 41.2% | M47 | 0.654 | > | 12 | 17 | 66.7% | 50.0% |
| | | ≤ | 5 | 14 | | | | | ≤ | 6 | 17 | | |
| M14 | 0.665 | > | 13 | 21 | 72.2% | 38.2% | M48 | 0.541 | > | 13 | 23 | 72.2% | 32.4% |
| | | ≤ | 5 | 13 | | | | | ≤ | 5 | 11 | | |
| M15 | 0.547 | > | 12 | 19 | 66.7% | 44.1% | M49 | 0.516 | > | 12 | 22 | 66.7% | 35.3% |
| | | ≤ | 6 | 15 | | | | | ≤ | 6 | 12 | | |
| M16 | 0.482 | > | 13 | 24 | 72.2% | 29.4% | M50 | 0.649 | > | 14 | 18 | 77.8% | 47.1% |
| | | ≤ | 5 | 10 | | | | | ≤ | 4 | 16 | | |
| M17 | 0.631 | > | 16 | 21 | 88.9% | 38.2% | M51 | 0.471 | > | 14 | 25 | 77.8% | 26.5% |
| | | ≤ | 2 | 13 | | | | | ≤ | 4 | 9 | | |
| M18 | 0.614 | > | 15 | 20 | 83.3% | 41.2% | M52 | 0.511 | > | 15 | 22 | 83.3% | 35.3% |
| | | ≤ | 3 | 14 | | | | | ≤ | 3 | 12 | | |
| M19 | 0.668 | > | 13 | 15 | 72.2% | 55.9% | M53 | 0.613 | > | 12 | 19 | 66.7% | 44.1% |
| | | ≤ | 5 | 19 | | | | | ≤ | 6 | 15 | | |
| M20 | 0.712 | > | 17 | 20 | 94.4% | 41.2% | M54 | 0.626 | > | 14 | 20 | 77.8% | 41.2% |
| | | ≤ | 1 | 14 | | | | | ≤ | 4 | 14 | | |
| M21 | 0.565 | > | 12 | 20 | 66.7% | 41.2% | M55 | 0.694 | > | 18 | 21 | 100.0% | 38.2% |
| | | ≤ | 6 | 14 | | | | | ≤ | 0 | 13 | | |
| M22 | 0.670 | > | 16 | 17 | 88.9% | 50.0% | M56 | 0.626 | > | 16 | 27 | 88.9% | 20.6% |
| | | ≤ | 2 | 17 | | | | | ≤ | 2 | 7 | | |
| M23 | 0.538 | > | 14 | 24 | 77.8% | 29.4% | M57 | 0.745 | > | 10 | 11 | 55.6% | 67.6% |
| | | ≤ | 4 | 10 | | | | | ≤ | 8 | 23 | | |

| | | | | | | | | | | | | | |
|---|---|---|---|---|---|---|---|---|---|---|---|---|---|
| M24 | 0.644 | > | 12 | 16 | 66.7% | 52.9% | M58 | 0.560 | > | 14 | 23 | 77.8% | 32.4% |
| | | ≤ | 6 | 18 | | | | | ≤ | 4 | 11 | | |
| M25 | 0.668 | > | 13 | 18 | 72.2% | 47.1% | M59 | 0.725 | > | 12 | 12 | 66.7% | 64.7% |
| | | ≤ | 5 | 16 | | | | | ≤ | 6 | 22 | | |
| M26 | 0.663 | > | 16 | 18 | 88.9% | 47.1% | M60 | 0.688 | > | 17 | 23 | 94.4% | 32.4% |
| | | ≤ | 2 | 16 | | | | | ≤ | 1 | 11 | | |
| M27 | 0.575 | > | 13 | 21 | 72.2% | 38.2% | M61 | 0.513 | > | 13 | 24 | 72.2% | 29.4% |
| | | ≤ | 5 | 13 | | | | | ≤ | 5 | 10 | | |
| M28 | 0.605 | > | 11 | 16 | 61.1% | 52.9% | M62 | 0.546 | > | 16 | 23 | 88.9% | 32.4% |
| | | ≤ | 7 | 18 | | | | | ≤ | 2 | 11 | | |
| M29 | 0.740 | > | 16 | 19 | 88.9% | 44.1% | M63 | 0.665 | > | 13 | 17 | 72.2% | 50.0% |
| | | ≤ | 2 | 15 | | | | | ≤ | 5 | 17 | | |
| M30 | 0.618 | > | 15 | 18 | 83.3% | 47.1% | M64 | 0.641 | > | 12 | 17 | 66.7% | 50.0% |
| | | ≤ | 3 | 16 | | | | | ≤ | 6 | 17 | | |
| M31 | 0.641 | > | 14 | 19 | 77.8% | 44.1% | M65 | 0.717 | > | 15 | 16 | 83.3% | 52.9% |
| | | ≤ | 4 | 15 | | | | | ≤ | 3 | 18 | | |
| M32 | 0.621 | > | 10 | 15 | 55.6% | 55.9% | M66 | 0.624 | > | 15 | 24 | 83.3% | 29.4% |
| | | ≤ | 8 | 19 | | | | | ≤ | 3 | 10 | | |
| M33 | 0.804 | > | 13 | 9 | 72.2% | 73.5% | M67 | 0.755 | > | 16 | 16 | 88.9% | 52.9% |
| | | ≤ | 5 | 25 | | | | | ≤ | 2 | 18 | | |
| M34 | 0.681 | > | 14 | 18 | 77.8% | 47.1% | | | | | | | |
| | | ≤ | 4 | 16 | | | | | | | | | |

**Supplementary Table 9.** List of PCR Primers (Illumina/Nextera i5 common adapter and i7 index adapters)

| Primer | Index | Sequences (5' to 3') |
|---|---|---|
| Ad1_noMX | | AATGATACGGCGACCACCGAGATCTACACTCGTCGGCAGCGTCAGATGTG |
| Ad2.1 | TAAGGCGA | CAAGCAGAAGACGGCATACGAGATTCGCCTTAGTCTCGTGGGCTCGGAGATGT |
| Ad2.2 | CGTACTAG | CAAGCAGAAGACGGCATACGAGATCTAGTACGGTCTCGTGGGCTCGGAGATGT |
| Ad2.3 | AGGCAGAA | CAAGCAGAAGACGGCATACGAGATTTCTGCCTGTCTCGTGGGCTCGGAGATGT |
| Ad2.4 | TCCTGAGC | CAAGCAGAAGACGGCATACGAGATGCTCAGGAGTCTCGTGGGCTCGGAGATGT |
| Ad2.5 | GGACTCCT | CAAGCAGAAGACGGCATACGAGATAGGAGTCCGTCTCGTGGGCTCGGAGATGT |
| Ad2.6 | TAGGCATG | CAAGCAGAAGACGGCATACGAGATCATGCCTAGTCTCGTGGGCTCGGAGATGT |
| Ad2.7 | CTCTCTAC | CAAGCAGAAGACGGCATACGAGATGTAGAGAGGTCTCGTGGGCTCGGAGATGT |
| Ad2.8 | CAGAGAGG | CAAGCAGAAGACGGCATACGAGATCCTCTCTGGTCTCGTGGGCTCGGAGATGT |
| Ad2.9 | GCTACGCT | CAAGCAGAAGACGGCATACGAGATAGCGTAGCGTCTCGTGGGCTCGGAGATGT |
| Ad2.10 | CGAGGCTG | CAAGCAGAAGACGGCATACGAGATCAGCCTCGGTCTCGTGGGCTCGGAGATGT |
| Ad2.11 | AAGAGGCA | CAAGCAGAAGACGGCATACGAGATTGCCTCTTGTCTCGTGGGCTCGGAGATGT |
| Ad2.12 | GTAGAGGA | CAAGCAGAAGACGGCATACGAGATTCCTCTACGTCTCGTGGGCTCGGAGATGT |
| Ad2.13 | GTCGTGAT | CAAGCAGAAGACGGCATACGAGATATCACGACGTCTCGTGGGCTCGGAGATGT |
| Ad2.14 | ACCACTGT | CAAGCAGAAGACGGCATACGAGATACAGTGGTGTCTCGTGGGCTCGGAGATGT |
| Ad2.15 | TGGATCTG | CAAGCAGAAGACGGCATACGAGATCAGATCCAGTCTCGTGGGCTCGGAGATGT |
| Ad2.16 | CCGTTTGT | CAAGCAGAAGACGGCATACGAGATACAAACGGGTCTCGTGGGCTCGGAGATGT |
| Ad2.17 | TGCTGGGT | CAAGCAGAAGACGGCATACGAGATACCCAGCAGTCTCGTGGGCTCGGAGATGT |
| Ad2.18 | GAGGGGTT | CAAGCAGAAGACGGCATACGAGATAACCCCTCGTCTCGTGGGCTCGGAGATGT |
| Ad2.19 | AGGTTGGG | CAAGCAGAAGACGGCATACGAGATCCCAACCTGTCTCGTGGGCTCGGAGATGT |
| Ad2.20 | GTGTGGTG | CAAGCAGAAGACGGCATACGAGATCACCACACGTCTCGTGGGCTCGGAGATGT |
| Ad2.21 | TGGGTTTC | CAAGCAGAAGACGGCATACGAGATGAAACCCAGTCTCGTGGGCTCGGAGATGT |
| Ad2.22 | TGGTCACA | CAAGCAGAAGACGGCATACGAGATTGTGACCAGTCTCGTGGGCTCGGAGATGT |
