# Supplementary material for: Chromatin accessibility of circulating CD8+ T cells predicts treatment response to PD-1 blockade in patients with gastric cancer
Source: Nat Commun. 2021 Feb 12;12:975. doi: 10.1038/s41467-021-21299-w (PMC7881150; doi:10.1038/s41467-021-21299-w)
Supplement: Supplementary file 3 — Descriptions of Additional Supplementary Files [file 41467_2021_21299_MOESM3_ESM.pdf]

Descriptions of Additional Supplementary Files

Supplementary Data 1

Description: Normalization control list

Supplementary Data 2

Description: Target list
